# Exosomes derived from adipose tissue-derived stem cells alleviated H_2_O_2_-induced oxidative stress and endothelial-to-mesenchymal transition in human umbilical vein endothelial cells by inhibition of the mir-486-3p/Sirt6/Smad signaling pathway

**DOI:** 10.1007/s10565-024-09881-6

**Published:** 2024-05-25

**Authors:** Yan Li, Yujie Xiao, Yage Shang, Chaolei Xu, Chao Han, Dahai Hu, Juntao Han, Hongtao Wang

**Affiliations:** Department of Burns and Cutaneous Surgery, Xijing Hospital, Air Force Medical University, 127 West Chang-Le Road, Xi’an, 710032 Shaanxi China

**Keywords:** Oxidative stress, EndoMT, ADSC-Exo, Mir-486-3p, Sirt6, Smad signaling pathway

## Abstract

Hypertrophic scar (HS) is characterized by excessive collagen deposition and myofibroblasts activation. Endothelial-to-mesenchymal transition (EndoMT) and oxidative stress were pivotal in skin fibrosis process. Exosomes derived from adipose tissue-derived stem cells (ADSC-Exo) have the potential to attenuate EndoMT and inhibit fibrosis. The study revealed reactive oxygen species (ROS) levels were increased during EndoMT occurrence of dermal vasculature of HS. The morphology of endothelial cells exposure to H_2_O_2,_ serving as an in vitro model of oxidative stress damage, transitioned from a cobblestone-like appearance to a spindle-like shape. Additionally, the levels of endothelial markers decreased in H_2_O_2_-treated endothelial cell, while the expression of fibrotic markers increased. Furthermore, H_2_O_2_ facilitated the accumulation of ROS, inhibited cell proliferation, retarded its migration and suppressed tube formation in endothelial cell. However, ADSC-Exo counteracted the biological effects induced by H_2_O_2_. Subsequently, miRNAs sequencing analysis revealed the significance of mir-486-3p in endothelial cell exposed to H_2_O_2_ and ADSC-Exo. Mir-486-3p overexpression enhanced the acceleration of EndoMT, its inhibitors represented the attenuation of EndoMT. Meanwhile, the target regulatory relationship was observed between mir-486-3p and Sirt6, whereby Sirt6 exerted its anti-EndoMT effect through Smad2/3 signaling pathway. Besides, our research had successfully demonstrated the impact of ADSC-Exo and mir-486-3p on animal models. These findings of our study collectively elucidated that ADSC-Exo effectively alleviated H_2_O_2_-induced ROS and EndoMT by inhibiting the mir-486-3p/Sirt6/Smad axis.

**Graphical Abstract:**

A schematic diagram summarizing the impact of ADSC-Exo on oxidative stress and endothelial-to-mesenchymal transition in endothelial cells was presented in this study. ADSC-Exo effectively alleviated the accumulation of ROS in endothelial cells induced by H_2_O_2_ and suppressed the pro-fibrotic function through modulation of the mir-486-3p/Sirt6/Smad signaling pathway. ADSC-Exo attenuated the up-regulation of mir-486-3p in endothelial cells exposure to H_2_O_2_, establishing a target relationship between mir-486-3p and Sirt6. Overexpression of Sirt6 inhibited the occurrence of endothelial-to-mesenchymal transition, thereby suppressing collagen deposition and myofibroblasts activity by the regulation of Smad2/3 phosphorylation. Consequently, this led to a reduction in hypertrophic scar formation.

Graphical Highlights

1. The occurrence and development of ROS and endothelial-to-mesenchymal transition promoted hypertrophic scar fibrosis;

2. H_2_O_2_ induced oxidative stress and EndoMT of endothelial cells, whereas ADSC-Exo ameliorated the process;

3. Mir-486-3p was crucial for H_2_O_2_-induced EndoMT and the improvement of ADSC-Exo;

4. Mir-486-3p directly targeted Sirt6 to facilitate EndoMT by regulating Smad signaling pathway.

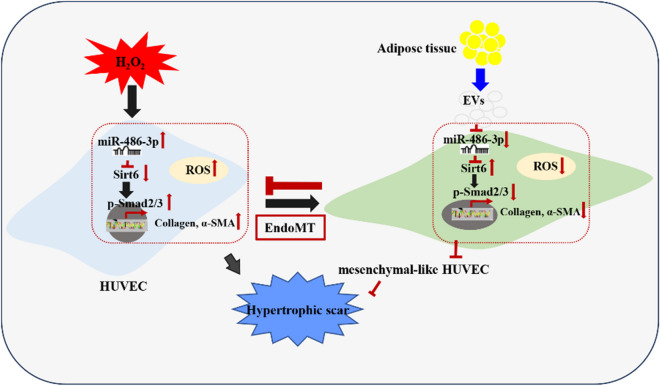

**Supplementary Information:**

The online version contains supplementary material available at 10.1007/s10565-024-09881-6.

## Introduction

Hypertrophic scar (HS), a fibrotic disease that needs urgent attention, is characterized by excessive collagen production and trans-differentiation of myofibroblasts following burn or traumatic injury. These processes often result in aesthetic disfiguration and functionary impairment for patients. The generation of myofibroblasts has been reported to occur through endothelial-to-mesenchymal transition (EndoMT) of endothelial cells, and substantial vascular remodeling is often observed in various fibrotic diseases prior to fibrosis initiation. However, the molecular mechanism underlying EndoMT function in hypertrophic scar formation have not been fully elucidated.

The process of EndoMT involves a phenotypic transition, endothelial cell loses the intrinsic properties and obtains mesenchymal-appearance features similar to fibroblasts under inflammation and oxidative stress conditions (Shenoy et al. 2016). Several recent studies had elucidated that EndoMT contributed pronouncedly to the conversion of mesenchymal cells population utilizing EC-lineage tracing in atherosclerosis, and EndoMT was engaged in pulmonary fibrosis caused by intratracheal administration of bleomycin (Yu et al. 2022). The purpose of our experiment was to explore the function of EndoMT in hypertrophic scar fibrosis. Hydrogen peroxide (H_2_O_2_), a stimulating factor of reactive oxygen species (ROS) generation, could rapidly deactivate nitric oxide and lead to oxidative stress damage in endothelial cell under the conditions of physiological homeostasis and inflammatory diseases (Iqbal et al. 2024; Morariu et al. 2008). The pathogenesis of pulmonary fibrosis (Dhaouafi et al. 2023), as well as NLRP3-mediated hepatocyte pyroptosis and subsequent liver fibrosis, was involved in the activation of oxidative stress (Xiao et al. 2023). TGFβ1-induced myofibroblast activation was associated with extracellular H_2_O_2_ production. The following experiments employed H_2_O_2_ to establish an in vitro model of oxidative stress injury to observe ROS-induced EndoMT of endothelial cells.

Adipose tissue stem cells (ADSCs) derived exosomes (Exo), as the crucial components in paracrine function, has been widely acknowledged, positioning them as critical mediators in cellular interaction (Lamichhane et al. 2015). The feature of parental cells would be preserved in the clinical application of exosomes, and exosomes internalized into the corresponding recipient cells prevent from fibrotic diseases, such as myocardial fibrosis, renal fibrosis and hepatic fibrosis (Sole et al. 2015; Cervio et al. 2015; Fiore et al. 2015). The study mainly kept the close attention to the role of ADSC-Exo in EndoMT of endothelial cells. In most cases, the primary mechanism by which ADSC-Exo exert their effects is implemented through modulating the expression of cellular miRNAs in recipient cells. MicroRNAs (miRNAs), as non-coding single-stranded RNAs, could antagonize target genes expression by binding to its 3′ UTR at post-transcriptional level (Iyer et al. 2017). The microRNA-486 is located on chromosome 8p11.21, originates from an intron within the *ANK1* locus and exerts the critical role in tumorigenesis. Two microRNAs, mir-486-5p and mir-486-3p, derive from opposite ends of pre-microRNA-486 hairpin structure (O'Brien et al. 2018), and the latter is overexpressed in erythroid cells. In the study, the comprehensive investigation of mir-486-3p function on EndoMT in endothelial cell necessitates further exploration.

Silencing information regulator 6 (Sirt6) is a histone III deacetylase. As a key epigenetic regulator, Sirt6 participates in multiple biological processes, including anti-inflammation, anti-oxidative stress, delaying aging and improving glucose/lipid metabolism (Zhang et al. 2018; Kim et al. 2019). Sirt6 alleviated phosphorylated mothers against decapentaplegic homolog (Smad) 2 levels as well as its nuclear transcription to suppress liver fibrosis in hepatic stellate cells, and in Sirt6 whole-body knockout mice, the up-regulation of TGFβ contributed to collagen deposition and ECM remodeling to facilitate cardiac, hepatic, renal and pulmonary fibrosis (Xiao et al. 2012; Maity et al. 2020).

In the study, the exacerbation of oxidative stress initially was shown in hypertrophic scar tissues, with the presence of endothelial-to-mesenchymal transition in dermal vasculature. Additionally, we established in vitro oxidative stress injury model to explore the impact of ADSC-Exo on ameliorating EndoMT in H_2_O_2_-induced human umbilical vein endothelial cells (HUVEC). Simultaneously, we confirmed that ADSCs exosomes promoted wound healing, alleviated collagen production, and reduced myofibroblast activity in a C57bl/6 mouse excisional model. Furthermore, we explored the specific molecular mechanism and validated mir-486-3p importance through directly regulating EndoMT. Subsequently, we identified the target regulatory relationship between mir-486-3p and Sirt6. Importantly, overexpression of Sirt6 mitigated EndoMT of endothelial cells, and mir-486-3p/Sirt6 axis strongly linked with Smad2/3 pathway. Collectively, our study elucidated the role of EndoMT in hypertrophic scar fibrosis and provided a novel strategy for clinical treatment.

## Methods

### Clinical ethics approval

Hypertrophic scar (HS), as well as adjacent normal skin (NS), atrophic scar (AS) and adipose tissues were harvested from patients who underwent plastic surgery in the department of burns and cutaneous surgery, Xijing Hospital, Xi'an. The work was performed in accordance with the Code of Ethic of the World Medical Association (Declaration of Helsinki). All participates were informed of the objective as well as process of this research, promised to supply their discarded tissues, and signed informed consent. The study was authorized by Ethics Committee of Xijing Hospital affiliated with Air Force Medical University (KY20202103-F-1).

### Cell culture

HUVEC were obtained from Cell Bank of Chinese Academy of Sciences (Shanghai, China), and cultured in endothelial cell medium (ECM, Sciencell #1001, San Diego, USA) supplemented with 5% FBS, 1% endothelial cell growth supplement and 1% penicillin–streptomycin in an incubator with 5% CO_2_ at 37℃. When HUVEC were grown to approximately 70%-80% confluence, stimulated with or without different concentrations of H_2_O_2_ (diluted with DMEM, 20 μM, 50 μM, 100 μM, 200 μM; 30% H_2_O_2,_ Tianjin Tianli Chemical Reagents Co., LTD) in the presence/absence of ADSC-Exo (20 μg/ml) for 24 h. Mir-486-3p mimics (100 nM/L), mir-486-3p inhibitors (200 nM/L) as well as their corresponding negative control (100 nM/L), or Sirt6 overexpression plasmid (OE-sirt6, EcoRI/BamHI cloning site) and empty vector of control plasmid pEX-1(Pgcmv/MCS/EGFP/Neo) (100 nM/L) (Genepharma, Shanghai) were transfected with lipofectamine 2000 Reagent kit (Invitrogen), the specific sequences shown in supplementary Table 1. HUVECs were simultaneously treated with 200 μM H_2_O_2_ and 20 μg/ml ADSC-Exo (20 μg/ml ADSC-Exo and 100 nM/L mir-486-3p mimics or 200 μM H_2_O_2_ and 200 nM/L mir-486-3p inhibitors) in a well of six-well plate. The mRNA samples were harvested after 24 h and the protein detection was performed after 48 h. The morphological change of HUVEC were observed by Olympus TH4-200 (IX70/71) (Table 1).

**Table 1:** The primer sequences of genes mentioned in the experiment

Gene	Species	Forward	Reward
Vimentin	hsa	5'-TGGATTCACTCCCTCTGGTTG-3'	5'-CGTGATGCTGAGAAGTTTCGTT-3'
α-SMA	hsa	5'-TGCTCCCAGGGCTGTTTTC-3'	5'-GATTCCTCTTTTGCTCTGTGCTT-3'
SM22α	hsa	5'-TTCCAGACTGTTGACCTCTTTGA-3'	5'-GCCCATCATTCTTGGTCACTG-3'
CD31	hsa	5'-CAGTGGAACTTTGCCTATTTCTTAC-3'	5'-ACGTCTTCAGTGGGGTTGTCT-3'
GAPDH	hsa	5'-GCACCGTCAAGGCTGAGAAC-3'	5'-TGGTGAAGACGCCAGTGGA-3'
CDH5	hsa	5'-GCCCTACCAGCCCAAAGTGT-3'	5'-CGTGTTATCGTGATTATCCGTGA-3'
Sirt6	hsa	5'-GTGGAAGAATGTGCCAAGTGTAA-3'	5'-CAGTCTAGGATGGTGTCCCTCAG-3'
Vimentin	mmu	5'-TCCAGAGAGAGGAAGCCGAA-3'	5'-TTCAAGGTCAAGACGTGCCA-3'
α-SMA	mmu	5'-GACAATGGCTCTGGGCTCTGTA-3'	5'-TTTGGCCCATTCCAACCATTA-3'
SM22α	mmu	5'-ACAAGGGTCCATCCTACGGC-3'	5'-GTTCACCAATTTGCTCAGAATCAC-3'
CD31	mmu	5'-GTTTCCCAAGCCGAAGTTAGA-3'	5'-TCTGATACTGCGACAAGACCGT-3'
GAPDH	mmu	5'-TGTGTCCGTCGTGGATCTGA-3'	5'-TTGCTGTTGAAGTCGCAGGAG-3'
CDH5	mmu	5'-ATTGGCCTGTGTTTTCGCAC-3'	5'-CACAGTGGGGTCATCTGCAT-3'
U6	hsa	5'-GGAACGATACAGAGAAGATTAGC-3'	5'-TGGAACGCTTCACGAATTTGCG-3'
mir-486-3p	hsa	5'-GGGGCAGCTCAGTACAGGAT-3'	
mir-486-3p mimics NC	hsa	5'-UCACAACCUCCUAGAAAGAGUAGA-3'	5'-UCUACUCUUUCUAGGAGGUUGUGA-3'
mir-486-3p mimics	hsa	5'-CGGGGCAGCUCAGUACAGGAU-3'	5'-AUCCUGUACUGAGCUGCCCCG-3'
mir-486-3p inhibitor NC	hsa	5'-UCUACUCUUUCUAGGAGGUUGUGA-3'	
mir-486-3p inhibitor	hsa	5'-AUCCUGUACUGAGCUGCCCCG-3'	
Lv3-mmu-mir-486-3p	mmu	5'-CGGGGCAGCTCAGTACAGGAT-3'	
Lv3-NC	mmu	5'-TTCTCCGAACGTGTCACGT-3'	
Smad2	hsa	5'-TTCAGTCTGTTAAGCCTACCACT-3'	5'-TGGGATACCTGGAGACGACC-3'
Smad3	hsa	5'-AGAGTTGAGGCGAAGTTTGGG-3'	5'-GTGAAAGGCAGGATGGACGA-3'
TGFβ1	hsa	5'-ATGGAGAGAGGACTGCGGAT-3'	5'-TAGTGTTCCCCACTGGTCCC-3'
Smad7	hsa	5'-CTGCAACCCCCATCACCTTA-3'	5'-TGGACAGTCAGTTGGTTTGAGAAA-3'

ADSCs were isolated as previously reported (Bai et al. 2010). Briefly, the shredded adipose tissues underwent enzymatic digestion with type I Collagenase (1 mg/ml, 0.1%, Sigma, SCR103) at 37℃ on the shaker for 50 min, then centrifuged and discarded the supernatants, cells were resuspended with ADSCs special medium (Ori cell HUXMD-90011, Cyagen, China) to T_25_ flashes.

### Flow cytometry

ADSCs at passages 3–5 were enzymatically dissociated using a 0.25% typsin-EDTA solution, centrifuged at 1000 rpm, 4℃, 5 min. Cells were washed with PBS and subsequently incubated with fluorescence-conjugated antibodies (CD105-PE, CD29-PE, CD34-FITC, CD44-PE, CD45-FITC, CD90-PE) for 30 min at 37℃ in the dark and examined by FACSAria™ III (BD Biosciences, USA).

### Adipogenic and osteogenic differentiation

Adipogenic and osteogenic differentiation were utilized to prove the multilineage of ADSCs. ADSCs at 80–90% confluence was incubated with the specific medium of adipogenic differentiation (OriCell, HUXMD-90031, Cyagen) for two weeks and osteogenic differentiation (OriCell, HUXMD-90021, Cyagen) for three weeks. Subsequently, the paraformaldehyde-prefixed ADSCs were identified with lipid droplet and calcium nodules under an optical microscope through Oil Red O Solution and Alizarin Red S. Images were obtained by Evos FL Auto2 (Invitrogen, Thermo Fisher Scientific).

### The acquisition, qualification and internalization of Exo

Exo was isolated as previously described (Logozzi et al. 2020). Briefly, the conditioned supernatants were subjected to centrifugate by 300 × g, 10 min, 4℃, followed by 2000 × g, 10 min, 4℃ and then 10000 × g, 30 min, 4℃. The obtained medium was filtered using a Millipore filter with a pore size of 0.22 μm, followed by ultracentrifugation by 100000 × g, 70 min, 4℃. Acquired pellets were dissolved with 26 ml PBS, then subsequently executed to another ultracentrifugation by 100000 × g, 70 min, 4℃. A Ti70 rotor of Beckman Coulter was utilized (Optima XPN-100 Ultracentrifuge). We ultimately resuspended exosomes with 200 μl PBS and stored at -80℃. BCA protein assays (Boster, Wuhan) was used to examine the concentrations of ADSC-Exo by infinite M200 PRO (TECAN). The concentration of exosome was standardized to 2 μg/μl on average.

ADSC-Exo were identified by the morphological and immunological detections. Immunoblotting of the specific markers (CD9 and CD63) determined the protein expressions, transmission electron microscope (TEM) represented the specific morphology, nanoparticle tracking analysis (NTA, ZetaView®system) detected the distributions of particle size of Exo, respectively. PKH26-labeled ADSC-Exo were performed to examine the internalization in HUVEC (Sigma-Aldrich). Briefly, Exo (diluted with 250 μl PBS) was diluted with 250 μl Diluent C, and then mixed with 2 μl PKH26 diluted in 250 μl Diluent C. The mixture was allowed to stand for 5 min to confirm a final PKH26 concentration of 1 × 10^−6^ M. Subsequently, it was neutralized with 5%BSA in PBS. The resulting mixture was centrifuged by 100000 × g, 70 min, 4℃. Finally, the obtained Exo was used to stimulate HUVEC.

### Immunohistochemistry staining

The paraffin-embedded sections were deparaffinized using dimethylbenzene, rehydrated with a series of ethanol dilutions, and subjected to antigen retrieval with Citrate-EDTA antigen retrieval solution (Beyotime, Shanghai). The activity of endogenous peroxidase was eliminated by utilizing 3% H_2_O_2_ at 37℃. Goat serum reduced the non-specific binding, the sections were then incubated with primary antibodies against α-SMA (CST, 1:300) and Col3 (Abcam, 1:200) at 4 °C overnight, incubated with the secondary antibody in the following day and visualized with DAB (SP Rabbit & Mouse HRP Kit, Cwbio, Beijing), followed by counterstaining nucleus with hematoxylin and observing under a light microscope (Evos FL Auto2, Invitrogen, Thermo Fisher Scientific).

### Immunofluorescence staining

HUVEC fixed in 4% paraformaldehyde (10^5^cells/well for 24-well plates) were permeabilized at room temperature with 0.1% TritonX-100, with 1% BSA blocking, subsequently incubation with primary antibodies against α-SMA (CST, 1:100), CD31 (Abcam, 1:100), VE-Cadherin (CST, 1:200), Vimentin (Proteintech, 1:100) and Ki67 (Proteintech, 1:100) overnight at 4℃. The paraffin-embedded sections were subjected to immunofluorescence staining, as previously mentioned, targeting α-SMA (Abcam, 1:200) and CD31 (CST, 1:200). The next day, cells or sections were incubated with secondary antibodies, labeled with either Cy3 or FITC (diluted with 1:50, Zhuangzhibio, Xi'an) at 37℃ for 1 h. Finally, counterstaining nucleus with DAPI (Boster, Wuhan), the pictures were captured using Evos FL Auto2 (Invitrogen, Thermo Fisher Scientific). The cytoskeleton staining of F-actin fibers was performed by incubating cells with Phalloidin (1:500, Abcam). The mean fluorescence intensity and immunofluorescence colocalization analysis were quantified by Fiji software (downloaded from http://fiji.sc).

### CCK8

Cell viability was detected by cell counting kit-8 (CCK8) assay (Meilunbio, Dalian). Briefly, HUVEC (10^4^ cells/100 μl) in 96-well plates were exposed to gradient concentrations of H_2_O_2_ (20, 50, 100, 200 μM) to last for 24 h. Addition of CCK8 reagent (10 μl/well) and incubation at 37℃ for 0.5 h or 1 h, the absorbance at OD450nm was measured using infinite M200 PRO (TECAN, Shanghai).

### Western blotting

Cellar lysates was centrifuged and detected the concentration of protein samples using the BCA protein assays (Beyotime, Shanghai). Subsequently, 25 μl of a 5 × loding buffer was pipetted to supernatants and degenerated at 100℃ for 10 min in a metal bath. The protein samples (30 μg) were separated using a 8–12% SDS-PAGE gel, subsequently electrophoretically transferred onto PVDF Transfer Membranes (0.45 μm pore size, Millipore, USA) at a voltage of 100 V for a duration of 40-90 min. Following transfer and blocking in defatted milk, the membranes were incubated with primary antibodies (dilution ratio/1:1000) against CD31(Proteintech, CST), VE-Cadherin (Boster, CST), α-SMA (Proteintech, CST), Nrf2 (Proteintech), Vimentin (Proteintech), SM22α (Boster), HO-1(Proteintech), ZO-1 (Proteintech), Sirt6 (CST), Nox4 (Proteintec), p-Smad2/3 (CST), Smad2/3 (CST), TGFβ1(Proteintech) and GAPDH (Zhuangzhi Bio) at 4℃ overnight (listed in Table 2). The next day, washing the membranes with TBST, incubation with HRP-conjugated secondary antibodies (Proteintech, dilution ratio/1:3000) at room temperature for 90 min, and visualization by utilizing an enhanced-chemiluminescence system (ECL Kit, Boster) on a ChemiDoc™ Imaging System (Bio-Rad). The quantitative analysis of protein intensity bands was performed by Image J software and normalized to GAPDH levels.

**Table 2:** Antibodies used in the study

Antibody	Species	Catalog (No.)	Manufacturer
NOX4	Rabbit	14,347–1-AP	Proteintech
CDH5	Rabbit	A02632-1	Boster
SM22α/TAGLN	Rabbit	A03962-2	Boster
ki67	Rabbit	27,309–1-AP	Proteintech
Vimentin	Rabbit	10,366–1-AP	Proteintech
CD31	Rabbit	11,265–1-AP	Proteintech
α-SMA	Rabbit	14,395–1-AP	Proteintech
ZO-1	Rabbit	21,773–1-AP	Proteintech
Sirt6 (D8D12)	Rabbit	#12,486	CST
α-SMA (D4K9N)	Rabbit	#19,245	CST
α-SMA	Mouse	ab7817	Abcam
VE-Cadherin (D87F2)	Rabbit	#72,026	CST
CD31(JC/70A)	mouse	ab9498	Abcam
ROS		S0033S	Beyotime
phospho-Smad2(Ser465/467)/Smad3(Ser423/425) (D27F4)	Rabbit	#8828	CST
Smad2/3 (D7G7)	Rabbit	#8685	CST
HO-1	Rabbit	10,701–1-AP	Proteintech
Nrf2	Rabbit	16,396–1-AP	Proteintech
CD31(PECAM-1)(D8V9E)	Rabbit	#77,699	CST
GAPDH	Rabbit	NC 021	Zhuangzhibio
TGFβ1	Rabbit	21,898–1-AP	Proteintech

### Transwell migration assays

The ability of migration was analyzed using an 8 μm pore size inserted in a 24-well plate (PI8P01250, Millipore), as previously described. Cells (1 × 10^4^ cells/well) were inoculated into the upper chamber. 500 μl medium, including the stimulation of H_2_O_2_ (200 μM), ADSC-Exo (20 μg/ml) and mir-486-3p (100 nM/mimics, 200 nM/ inhibitors), was transferred to the lower chamber. Experiencing an 8-h incubation, fixation and subsequently staining with crystal violet (Heart Biological technology, Xi'an), HUVEC distributed in the inner layer gently wiped off with cotton swabs. The migrated endothelial cells were analyzed by Fiji software (http://fiji.sc).

### Matrigel assay

The yellow tips were pre-cooled, and a 12/24 well plate along with Matrigel matrix (356254, Corning, BD Biosciences) was prepared in advance by storing them in a refrigerator at 4℃ for one day. Each well of the 24-well plates was uniformly coated with 200-300 μl of cold Matrigel, followed by incubation at 37℃ for half an hour to allow Matrigel solidification. Meanwhile, HUVEC were enzymatically dissociated using a 0.25% Typsin-EDTA solution. Subsequently, 2 × 10^5^cells/well of HUVEC were exposed to 200 μM H_2_O_2_, 20 μg/ml ADSC-Exo, 100 nM mir-486-3p mimics and 200 nM mir-486-3p inhibitors. Photographic documentation was performed at time points of 2 h, 4 h, 6 h to observe the formation of tubular structures. The quantity analysis of branch points and the length of tube was quantified through Image J software (https://imagej.net/ij/) with blood vessels plug-in.

### qPCR

The concentration of RNA lysates was measured (Epoch, BioTek instruments, Inc.). For reverse transcription, 500 ng RNA for synthesizing cDNA with Prime Script™ RT Master Mix kit (Takara, Japan), while 800 ng miRNAs with miRNA 1st strand cDNA synthesis kit (Accurate biology, Changsha) on a C1000™ Thermal Cycler system (Bio-Rad). The amplification of cDNA was executed with SYBR Premix Ex TaqTM II kit (Takara, Japan) or UltraSYBR mixture (Cwbio, Beijing) on a CFX96™ Real-Time System (Bio-Rad). Genes expression was confirmed by performing every reaction in triplicate, with regarding GAPDH as an inner control. U6 was utilized as an internal normalization for mir-486-3p. The primer pairs appeared in the experiment were documented in Table 1, with each experiment being replicated thrice. Relative quantification was conducted following the ΔΔCT method, and results were represented in the linear form using the formula 2-^ΔΔCT^.

### miRNAs-sequence

The enrichment of 18–30nt RNA molecules by polyacrylamide gel electrophoresis (PAGE), 36-44nt RNAs by the addition of 3'adapters and the subsequent administration of 5' adapters, then the PCR amplification of the ligation products through reverse transcription, the enrichment of 140-160 bp size of PCR products were generated a cDNA library and sequenced using Illumina Novaseq6000 by Gene Denovo Biotechnology Co. (Guangzhou, China). The differential expression analysis of miRNAs was performed by edgeR software between two different groups or samples. miRNAs were identified with a fold change ≥ 1.5 and *p* value < 0.05 in a comparison as significant differentially expressed miRNAs. The raw miRNA sequencing data of this study had been deposited in the NCBI Sequence Read Archive (SRA) database under the accession code PRJNA952693 (https://ncbi.nlm.nih.gov/).

### Luciferase reporter assay

The RNAhybrid database predicted the binding sequences of mir-486-3p and Sirt6. Sirt6 3'UTR containing wild-type (WT) or mutant (Mut) binding site of human mir-486-3p were designed and synthesized by GenePharma (Shanghai, China). 293 T were co-transfected with the corresponding plasmids and human mir-486-3p mimics/mimics-NC or inhibitors/inhibitors-NC with Lipofectamine 2000 (Invitrogen). To construct of luciferase reporter gene vector containing Sirt6 promotor, the full-length Sirt6 promotor containing wild or mutant type was respectively cloned into pGL3-basic vectors (Genecreate, Wuhan, China), and co-transfected with or without Sirt6 overexpression vector. After 48 h of incubation, the activities of firefly and Renilla luciferase were measured using the Dual Luciferase Reporter Assay Kit (Promega, Madison, WI, USA). The binding sequences of miRNA and target gene were shown as follows, hsa-miR-486-3p: CGGGGCAGCTCA GTACAGGAT; Sirt6-WT: CTGTGCTCCAGGCCAGGGGTTACACCTGCCCT; Sirt6-MT: TCACATCCCAGGCCAGAAATTA CACTCATTTC.

### Animal experiments

C57BL/c male mice (Six- to eight-week-old) were acquired from Experimental Animal Center of Air Force Medical University. All protocols and experiments were authorized by Laboratory Animal Welfare and Ethics Committee of the Air Force Medical University (Approval number: 20231002) and executed in strict accordance with the requirements of above-mentioned institutions (Xi'an, China). The animals were randomly allocated into the following four groups: Ctrl/PBS groups, Exo group (70 μg dilution into 100 μl PBS per mouse), Exo + mir-486-3p mimics NC groups and Exo + Lv3-mmu-mir-486-3p mimics groups (1 × 10^9^TU/ml virus titer in PBS, Genepharma, Shanghai). The mice were anesthetized with isoflurane, the hair on their dorsal surface was removed, and a 1 cm in a diameter of full-thickness skin defect model was created and splinted with a silicone ring possessing super adhesive properties. ADSC-Exo and the lentivirus were administrated into the wound through subcutaneous injection with a 27-gauge needle to last for either 5 days or 3 days, respectively. The wounds were documented on days 3, 5, 7, 10, 14. Following a two-week period, the wound tissues of euthanized mice were collected for the follow-up histological staining. Each group consisted of a minimum of six mice.

The paraffin-embedded samples sections were utilized for H&E and Masson trichrome staining (Jiancheng Bioengineering Institute, Nanjing) to assess pathological change and collagen deposition according to the manufacturer's instructions. The total mRNA and protein of wound tissues were extracted using a tissue lyser servicer, and centrifuged. The supernatants were utilized for subsequent concentration determination of mRNA and protein levels of corresponding molecules.

### ROS assay

We detected oxidative stress levels using the Reactive Oxygen Species Assay Kit (Beyotime Biotechnology, Shanghai). 70–80% confluence of HUVEC in six-well plates were stimulated with H_2_O_2_, ADSC-Exo and catalase. After 24 h, cells were treated with tryptic digestion, and stained with DCFH-DA (10μΜ/L) at 37℃ on the shaker in the absence of light. Following another round of PBS, labeled HUVEC in 300 μl of serum-free medium was subjected to flow cytometry analysis using an excitation wavelength of 488 nm and an emission wavelength of 525 nm.

### Statistical analysis

The data in the study were analyzed utilizing GraphPad8 Prism software. Each operation was repeated at least three times, and the results were presented as mean ± standard error of the mean. Statistical comparisons between two groups were performed using Student′s T test. *p* < *0.05* was considered statistically significant.

## Results

### The occurrence of ROS and EndoMT has been observed in hypertrophic scar

**Fig. 1:**
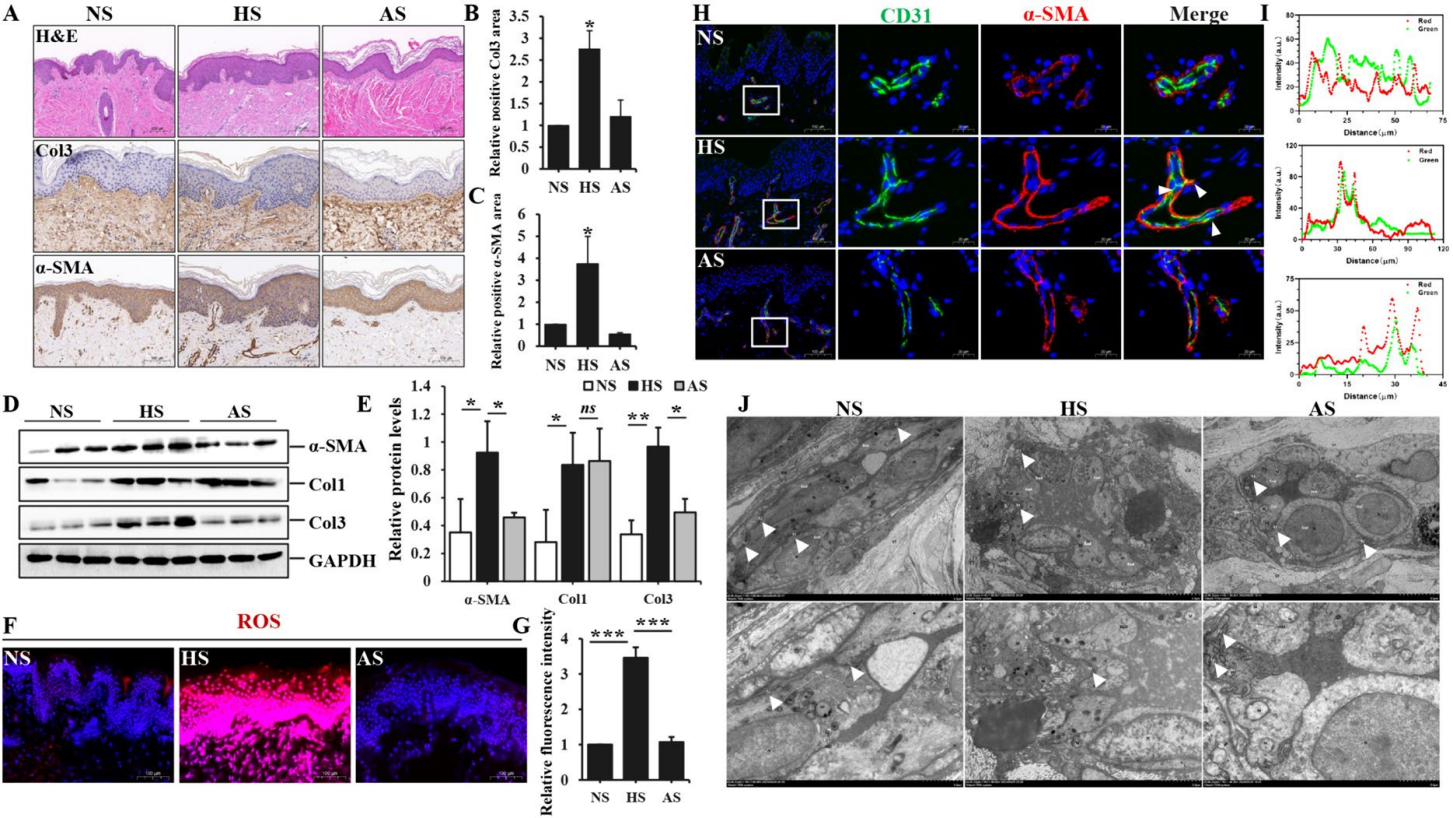
The occurrence of oxidative stress and EndoMT in dermal vasculature of hypertrophic scar. **A** Routine Hematoxylin–Eosin staining and immunohistochemistry staining for Col3 and α-SMA in normal skin (NS), hypertrophic scar (HS) and atrophic scar (AS), Scale bars = 200 μm, 100 μm. **B** the quantitative analysis of Col3 performed by immunohistochemical staining. **C** the quantitative analysis of α-SMA immunohistochemical staining. **D** western blot analysis of Col1, Col3 and α-SMA using GAPDH as internal control. **E** the quantitative analysis of WB bands was determined based on the results obtained from three repeated experiments. **F** ROS levels measured by immunofluorescence staining in NS, HS and AS, Scale bars = 100 μm. **G** the quantitative analysis of mean fluorescence intensity of ROS in HUVEC stimulated with different concentrations of H_2_O_2_ and 20 μg/ml ADSC-Exo. **H** the dual immunofluorescence staining of antibodies against CD31 (Red, endothelial marker) and α-SMA (FITC, fibroblast marker) in NS, HS and AS, the colocalization was visualized in yellow color and nuclei were counterstained with DAPI (blue). Scale bars = 100 μm, 20 μm. **I** the qualitative analysis of colocalization immunofluorescence staining (CD31 and α-SMA) measured by Fiji software (downloaded by https://fiji.sc). **J** The TEM images shown the presence of well-defined tight junction among endothelial cells in NS, HS and AS. Scale bars = 5 μm, 2 μm. Data represented the mean ± SD of triplicates. ^*^p < *0.05*, ^**^p < 0.01, ^***^p < 0.001, *ns*, non-significant

Resident fibroblasts proliferation and endothelial cells through endothelial-to-mesenchymal transition resulted in the large accumulation of fibroblasts in visceral fibrosis (Yu et al. 2022; Li et al. 2022). In hypertrophic scar, we initially confirmed the overexpression of collagen and α-SMA through immunohistochemistry staining and immunoblot analysis (Fig. 1A, D). There were statistically significant differences between HS groups and the other two groups, except for Col1 expression between HS and AS (Fig. 1B-C, E). Additionally, the immunofluorescence staining revealed a remarkable increase of ROS levels in HS, indicating the activation of oxidative stress (Fig. 1F-G). The colocalization staining of CD31 (an endothelial marker) and α-SMA (a fibrotic marker) was further employed to validate the occurrence of EndoMT in HS. As depicted in Fig. 1H, the endothelium layer of dermal vascular in HS exhibited a higher presence of yellow-fluorescent signals compared to NS and AS, suggesting that endothelial cells underwent trans-differentiation into mesenchymal-like cells to promote fibrosis, a process known as EndoMT. The transitional EndoMT cells in HS exhibited simultaneous expression of CD31 and α-SMA, indicating a potential role in enhanced permeability. A large proportion of fluorescent intension showed the same trends between CD31/red and α-SMA/green in HS (Fig. 1I). Meanwhile, the transmission electron microscope revealed that the electron density of vascular tight junctions (TJs) was remarkably lower in HS, the gap between two endothelial cells appeared more pronounced as well (Fig. 1J). Our findings collectively provided evidence for the occurrence of EndoMT in HS and its correlation with oxidative stress.

### H_2_O_2_ activated and facilitated the transition of endothelial cells into a fibroblasts-like appearance

**Fig. 2:**
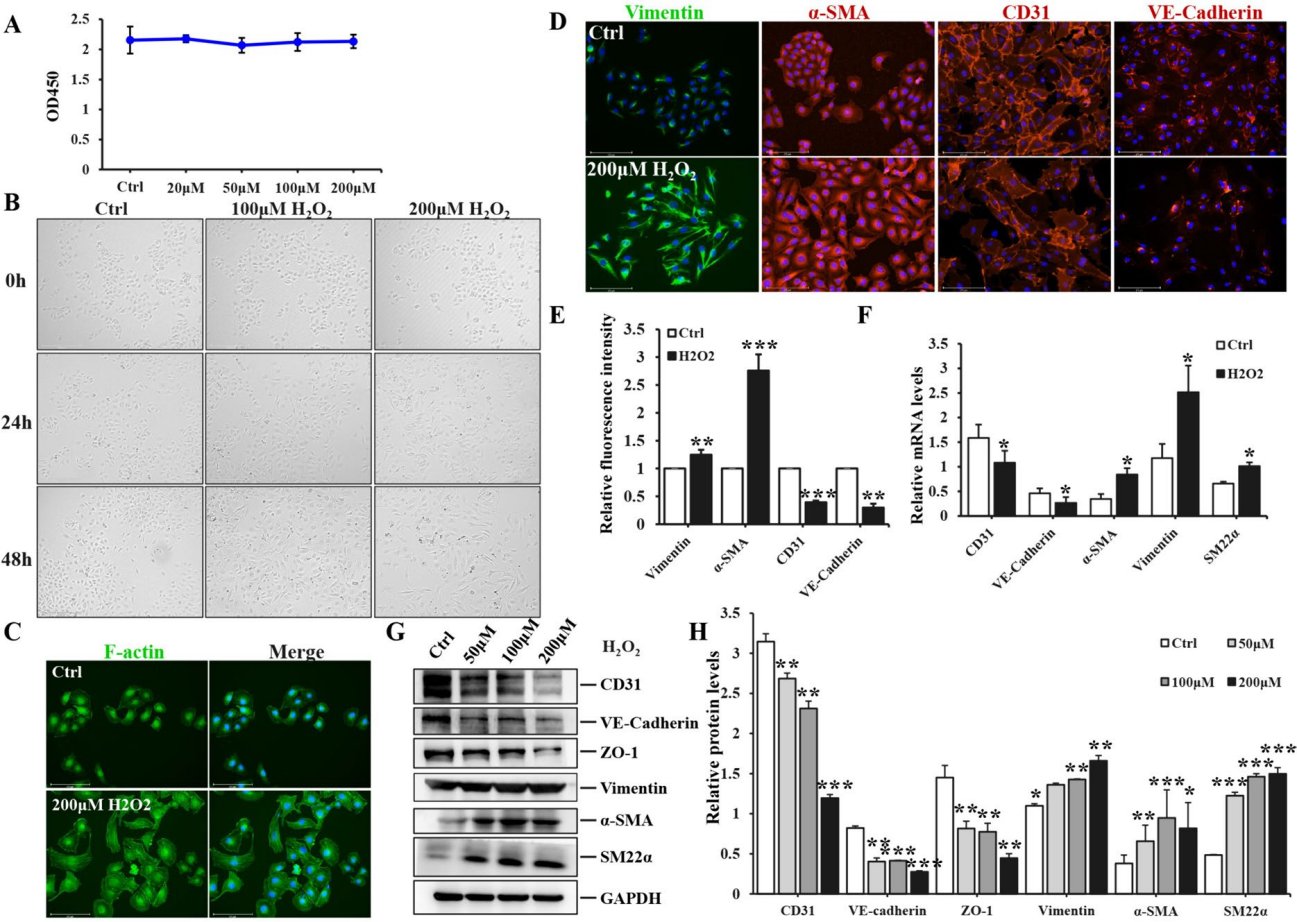
The induction of EndoMT in HUVEC in vitro by H_2_O_2_. **A** the OD450 absorbance in HUVEC exposed to different concentrations of H_2_O_2_ (20, 50, 100, 200 μM). **B** the morphological changes of HUVEC were observed under a light microscope after stimulation with 100 μM H_2_O_2_ or 200 μM H_2_O_2_ for 24 h and 48 h, Scale bars = 650 μm. **C** immunofluorescence staining of F-actin was performed in HUVEC exposed to either PBS or 200 μM H_2_O_2,_ Scale bars = 275 μm. **D** immunofluorescence staining of Vimentin, α-SMA, CD31 and VE-Cadherin in HUVEC treated with PBS or 200 μM H_2_O_2,_ Scale bars = 275 μm. **E** the quantitative analysis of mean fluorescence intensity of aforementioned proteins. **F** qPCR analysis was performed to assess the mRNA levels of CD31, VE-Cadherin, Vimentin, α-SMA and SM22α in HUVEC stimulated with PBS or 200 μM H_2_O_2_. **G** immunoblot analysis was performed to assess the protein expression of CD31, VE-Cadherin, ZO-1, Vimentin, α-SMA and SM22α in HUVEC exposed to PBS, 50 μM, 100 μM or 200 μM H_2_O_2_. **H** The intensity of protein bands was quantified. Each experiment was conducted in triplicate. Data represented the mean ± SD of triplicates. ^*^p < *0.05*, ^**^p < 0.01, ^***^p < 0.001

Serving as an in vitro model for oxidative stress, H_2_O_2_ could induce the initiation and progression of EndoMT in HUVEC. We exposed HUVEC to various concentrations of H_2_O_2_ (20 μM, 50 μM, 100 μM, 200 μM) for 24 h in order to assess cell viability. 200 μM was eventually selected as the optimal stimulation concentration for the following experiments (Fig. 2A). The morphology of HUVEC exposed to 100 μM or 200 μM H_2_O_2,_ as depicted in Fig. 2B, underwent a prominent transformation from the typical clusters and cobblestone-like appearance of endothelial cells to a spindle-shaped, elongated fibroblast-like phenotype of mesenchymal cells. This change suggested that the administration of H_2_O_2_ could induce EndoMT. Similarly, the cytoskeleton F-actin of HUVEC exposed to H_2_O_2_ also exhibited a pronounced morphological alteration through immunofluorescence staining, as previously mentioned (Fig. 2C). Furthermore, the fluorescence expression of profibrotic markers (Vimentin and α-SMA) was significantly elevated in HUVEC exposed to 200 μM H_2_O_2_, whereas the fluorescence intensity of CD31 and VE-Cadherin was reduced following H_2_O_2_ treatment (Fig. 2D), the difference for mean fluorescence intensity of two groups was statistical significance (Fig. 2E). These findings were further verified by qPCR and WB analysis, demonstrating a significant up-regulation of fibrotic molecules (Vimentin, α-SMA and SM22α) in H_2_O_2_-induced HUVEC (50 μM, 100 μM, 200 μM), as well as a dose-dependent down-regulation of endothelial markers (Fig. 2F-H). Additionally, cell adherent junction protein ZO-1 was also dramatically reduced. There were statistically significant differences in different concentrations of H_2_O_2_ groups compared to control group. These results illustrated the successful induction of EndoMT in HUVEC by H_2_O_2_.

### The isolated ADSC-Exo were identified and tracked in endothelial cells

**Fig. 3:**
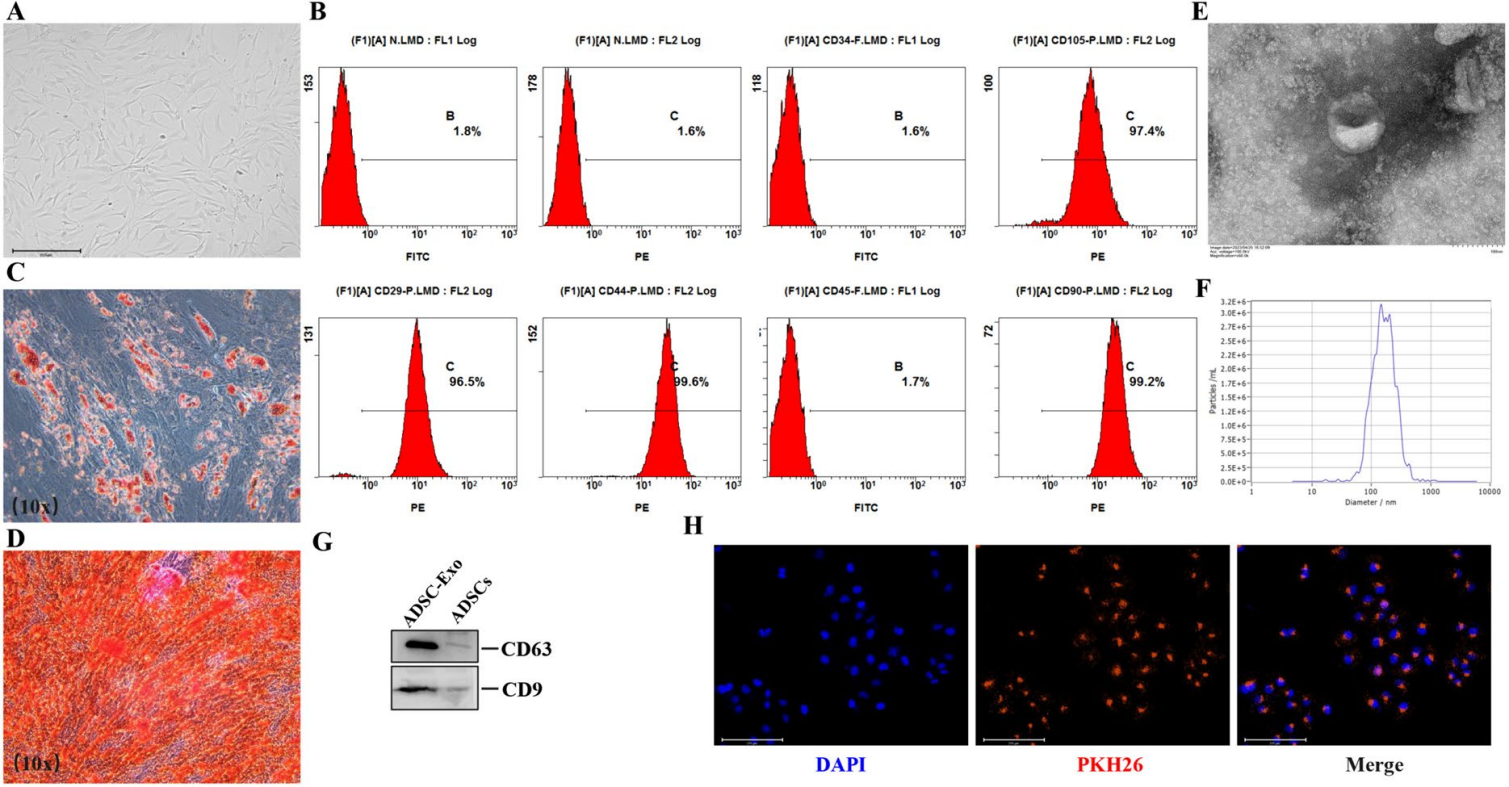
The identification and intercellular localization of ADSC-Exo in HUVEC. **A** the morphological characteristics of ADSCs observed under a light microscope, Scale bars = 650 μm. **B** the surface markers of ADSCs were analyzed by flow cytometry (CD29-PE, CD34-FITC, CD44-PE, CD45-FITC, CD73-PE, CD90-PE, CD105-PE). **C**-**D** the adipogenic and osteogenic differentiation of ADSCs were assessed by staining with Oil Red O and Alizarin Red S, respectively. Higher magnification (10 ×). **E** the identification of ADSC-Exo by TEM, Scale bars = 100 nm. **E** NTA analysis of the dimensions of ADSC-Exo. **F** immunoblot analysis was conducted to examine the specific markers of ADSC-Exo (CD9, CD63). **G** the internalization of PKH26-labeled ADSC-Exo in HUVEC. Scale bars = 275 μm

The therapeutic effect of ADSCs on attenuating visceral and skin fibrosis is a widely accepted fact. The isolated human ADSCs exhibited a typical fibroblast-like, spindle-shaped morphology and showed the capacity of adipogenic and osteogenic differentiation, as evidenced by the appearance of lipid droplets through Oil Red O staining and calcium nodules detected using Alizarin Red S staining (Fig. 3A, C-D). ADSCs exhibited positive expression of surface markers associated with mesenchymal stem cells, including CD105 (97.4%), CD29 (96.5%), CD44 (99.6%), CD90 (99.2%), and negative expression of hematopoietic markers (CD34-1.6%, CD45-1.7%) as determined by immunophenotypic analysis (Fig. 3B). The aforementioned results were consistent with the typical characteristics of ADSCs. ADSC-Exo, serving as cellular interaction and an effective carrier for paracrine function, was deemed to possess similar properties to their parental cells (ADSCs). Subsequently, we employed TEM, NTA and WB techniques to identify ADSC-Exo. As shown in Fig. 3E-G, the exosomes presented a bilayer lipid membrane structure with characteristic teacup-like morphology. Besides, the protein expression of CD9 and CD63 was up-regulated in ADSC-Exo, and the mean vesicle size measured 179 nm. These results directly verified that the isolated nanoparticles were exactly exosomes. Then, we used PKH26 to label the exosomes for observing their internalization by HUVEC, and ADSC-Exo could be efficiently endocytosed by HUVEC (Fig. 3H).

### ADSC-Exo alleviated H_2_O_2_-induced EndoMT in HUVEC

**Fig. 4:**
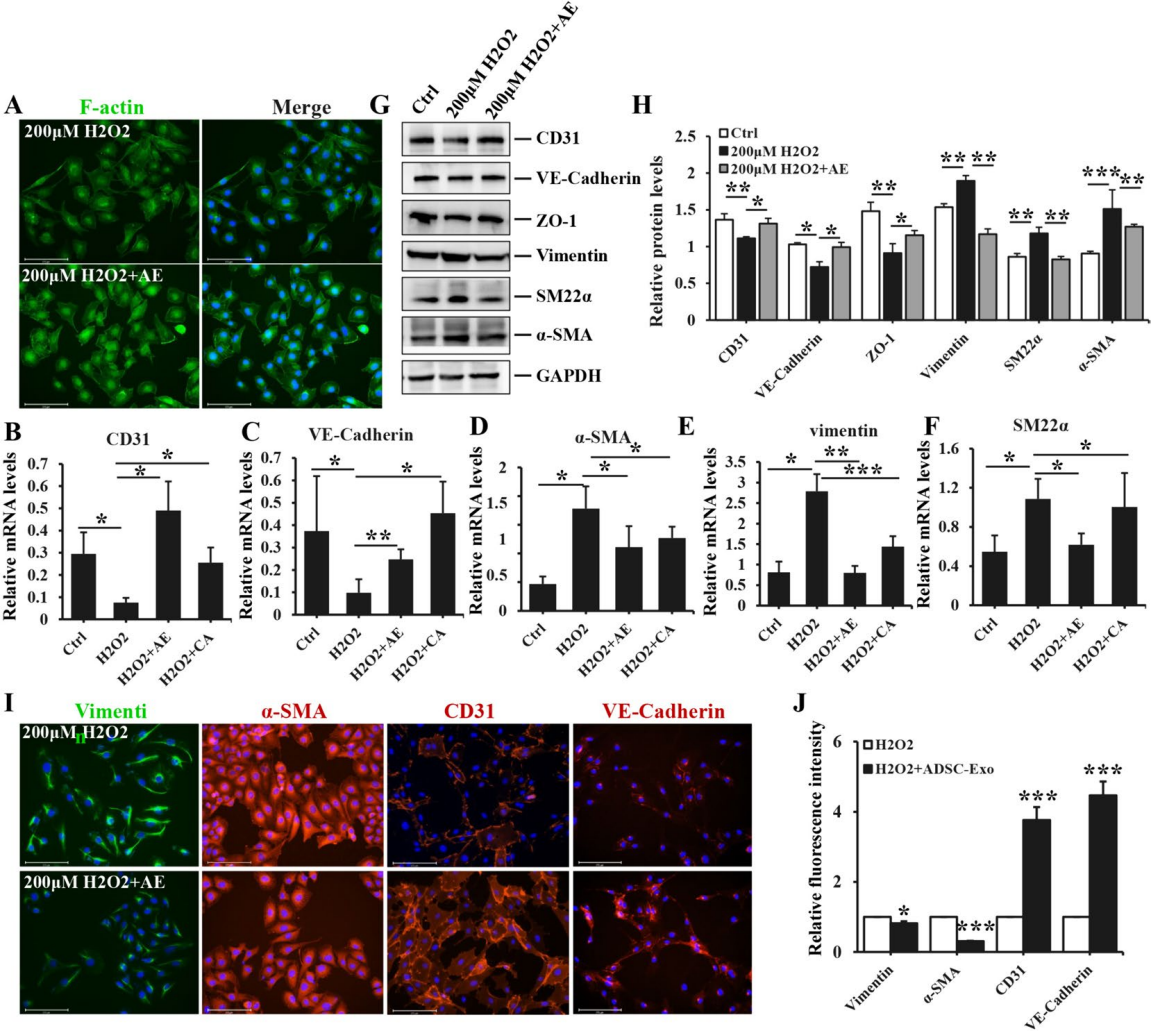
The impact of ADSC-Exo on H_2_O_2_-induced EndoMT in HUVEC. **A** immunofluorescence staining of F-actin was performed in HUVEC treated with 200 μM H_2_O_2_ or combination with 20 μg/ml ADSC-Exo. Scale bars = 275 μm. **B**-**F** the mRNA levels of CD31, VE-Cadherin, α-SMA, Vimentin and SM22α were quantified by qPCR analysis in HUVEC exposed to PBS, 200 μM H_2_O_2_ or 200 μM H_2_O_2_ + 20 μg/ml ADSC-Exo. **G** WB analysis was performed to assess the protein levels of CD31, VE-Cadherin, ZO-1, Vimentin, SM22α, and α-SMA in HUVEC exposed to PBS, 200 μM H_2_O_2_ or 200 μM H_2_O_2_ + 20 μg/ml ADSC-Exo. **H** the quantitative analysis of protein bands intensity. **I** immunofluorescence staining of Vimentin, α-SMA, CD31 and VE-Cadherin were performed in HUVEC stimulated with 200 μM H_2_O_2_ or combination with 20 μg/ml ADSC-Exo. Scale bars = 275 μm. **J** the quantitative analysis of mean fluorescence intensity of aforementioned proteins. The experiment was conducted in triplicate. Data represented the mean ± SD of triplicates. ^*^p < *0.05*, ^**^p < 0.01, ^***^p < 0.001

The function of Exo antagonizing fibrosis was utilized to investigate its role in H_2_O_2_-induced EndoMT. Immunofluorescence staining of F-actin revealed that ADSC-Exo significantly reversed the morphological change of endothelial cells exposure to H_2_O_2_ from fibroblast-like to cobblestone appearance (Fig. 4A). Additionally, treatment with ADSC-Exo resulted in a reduction of mRNA expression of α-SMA, Vimentin and SM22α in H_2_O_2_-induced HUVEC, and simultaneously increased the levels of CD31 and VE-Cadherin. These findings suggested that ADSC-Exo exhibited significant inhibitory effects on H_2_O_2_-induced EndoMT of HUVEC. It was noteworthy that catalase was employed as a positive control (Fig. 4B-F), ADSC-Exo treatment group showed statistically significant differences. Moreover, the aforementioned protein expression were consistent with mRNA levels. Additionally, ZO-1 expression was up-regulated after ADSC-Exo treatment, as shown in Fig. 4G-H. These findings suggested that the administration of ADSC-Exo had a protective function in EndoMT of HUVEC to some extent, with notable statistical differences observed between two groups. More importantly, Exo pronouncedly ameliorated the fluorescence intensity of α-SMA and Vimentin in HUVEC exposure to H_2_O_2_, but the fluorescence intensity of CD31 and VE-Cadherin exhibited a significant increase following ADSC-Exo treatment (Fig. 4I), indicating the potential anti-EndoMT effect of ADSC-Exo on HUVEC exposure to H_2_O_2_. The mean fluorescence intensity between two groups was of statistical significance (Fig. 4J).

### The treatment of ADSC-Exo alleviated H_2_O_2_-induced oxidative stress and improved the biological function of HUVEC

**Fig. 5:**
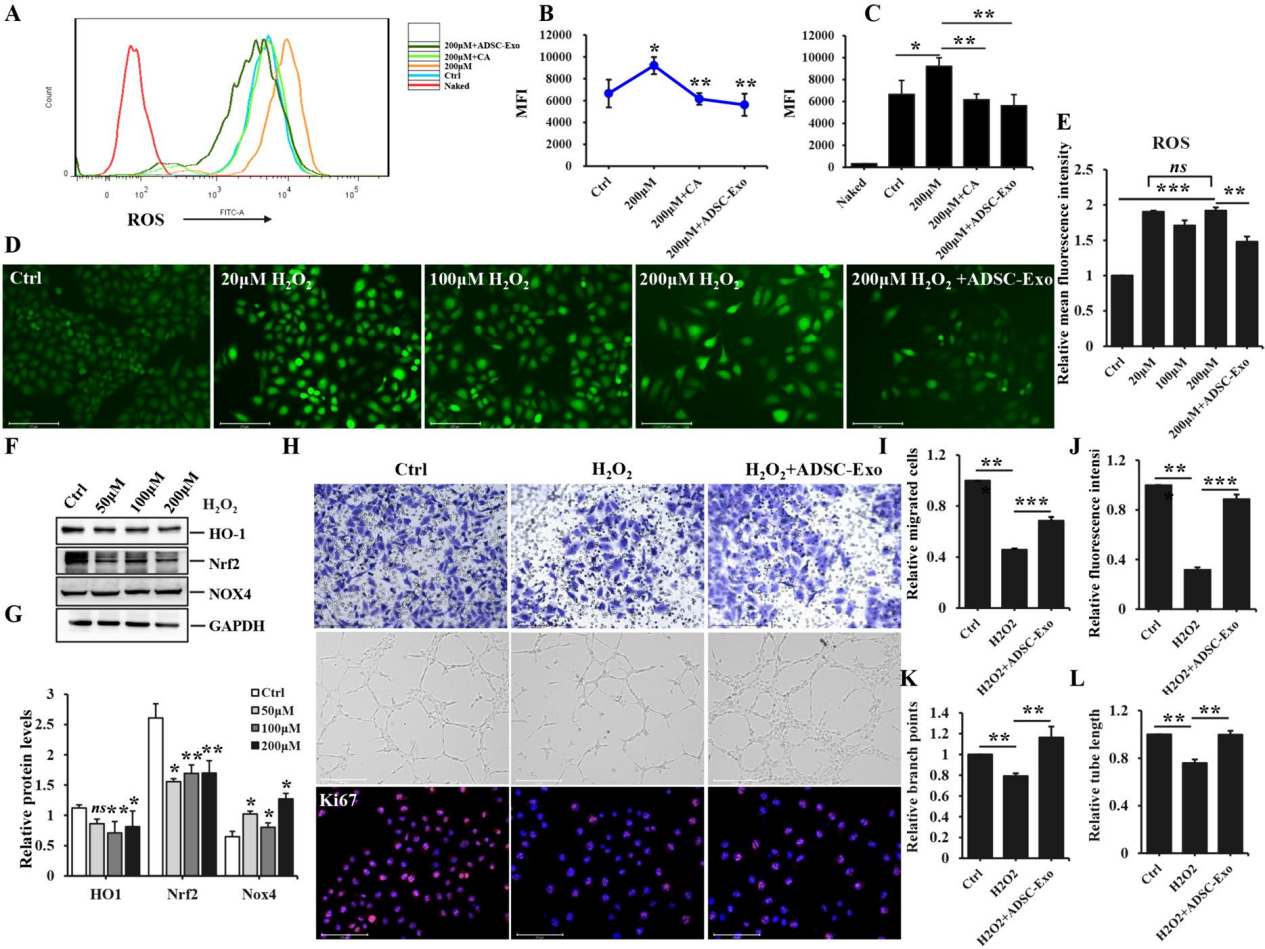
The effect of H_2_O_2_ and ADSC-Exo on ROS generation and HUVEC biological function. **A** flow cytometry analysis was performed to evaluate the levels of ROS in HUVEC exposed to H_2_O_2_, ADSC-Exo and catalase. **B**-**C** the quantitative analysis of ROS levels. **D** the immunofluorescence expression of ROS in HUVEC exposed to different concentrations of H_2_O_2_ (20, 50, 100, 200 μM) and 20 μg/ml ADSC-Exo. Scale bars = 275 μm. **E** the quantification of mean fluorescence intensity of ROS in different groups. **F** WB analysis was conducted to examine the levels of oxidative stress-related proteins in HUVEC exposed to different concentrations of H_2_O_2_ (50, 100, 200 μM)_._ **G** the quantification of protein bands intensity. **H** the proliferation, migration and tube formation in HUVEC exposed to H_2_O_2_ and ADSC-Exo were assessed by immunofluorescence staining of Ki67, crystal violet staining and Matrigel assays, respectively. Scale bars = 650 μm, 500 μm and 275 μm. **I** the quantification of migrated cells in transwell assays. **J** the quantitative analysis of mean immunofluorescence intensity of Ki67 in HUVEC exposed to H_2_O_2_ and ADSC-Exo. **K**-**L** the quantity analysis of branch points and tube length in tube formation assays. Data represented the mean ± SD of triplicates. ^*^p < *0.05*, ^**^p < 0.01, ^***^p < 0.001, *ns*, non-significant

The significantly up-regulated levels of ROS in hypertrophic scar tissues prompted us to investigate the impact of H_2_O_2_ on oxidative stress of HUVEC. Flow cytometry analysis demonstrated H_2_O_2_ dramatically induced the generation of ROS. However, both ADSC-Exo and catalase (a scavenger of H_2_O_2_) remarkably alleviated intracellular ROS induced by H_2_O_2_, thereby mitigating oxidative stress in HUVEC, the two groups exhibited significant statistical differences in terms of their ability to suppress the deleterious effects of ROS and modulate cellular physiology (Fig. 5A-C). Meanwhile, there was a noticeable elevation in the levels of ROS through immunofluorescence analysis in HUVEC exposed to H_2_O_2_ (20 μM, 100 μM, 200 μM) (Fig. 5D), showing significant differences between experimental groups and control group (Fig. 5E). Nox4 protein expression in HUVEC stimulated with H_2_O_2_ was increased, whereas HO-1 and Nrf2 protein levels were decreased, representing statistically significant differences between two groups except for HO-1 expression (*Ctrl vs 50 μM H*_*2*_*O*_*2*_* group*) (Fig. 5F-G). The aforementioned observations indicated H_2_O_2_ could activate oxidative stress and lead to EndoMT of endothelial cells. More importantly, we observed that ADSC-Exo effectively restored the proliferation, migration and tube formation of HUVEC exposed to H_2_O_2_ (Fig. 5H), presenting much number of migrated endothelial cells, stronger fluorescence intensity of ki67, more branch points and longer tube length with a statistical difference (Fig. 5I-L), thereby highlighting the crucial function of ADSC-Exo in defending against oxidative stress-induced EndoMT.

### The mir-486-3p was differentially expressed in H_2_O_2_-induced HUVECs with or without ADSC-Exo

**Fig. 6:**
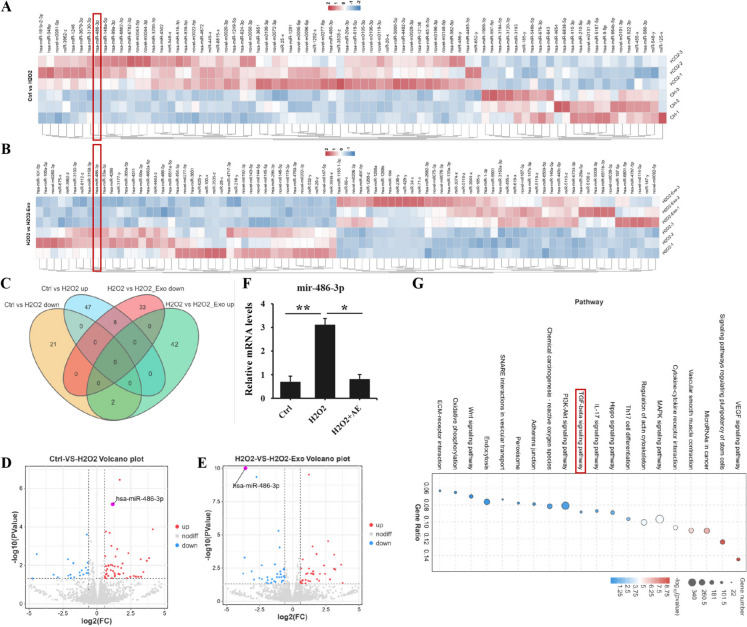
The analysis and interpretation of miRNA sequencing data. **A**-**B** heatmap analysis was conducted to visualize the clustering analysis of differentially expressed miRNAs between the two groups (Ctrl *vs* 200 μM H_2_O_2_ group or 200 μM H_2_O_2_ *vs* 200 μM H_2_O_2_ + 20 μg/ml ADSC-Exo group). **C** the Venn diagram illustrated the number of differentially expressed miRNAs that overlapped between the two groups. **D** the volcano plots revealed the differentially expressed miRNAs, with up-regulated ones shown in red and down-regulated ones shown in blue, for each comparison between the two groups (Ctrl *vs* 200 μM H_2_O_2_ group or 200 μM H_2_O_2_ *vs* 200 μM H_2_O_2_ + 20 μg/ml ADSC-Exo group), the distinct marking of mir-486-3p was clearly indicated by the arrows. **E** qPCR analysis was performed to evaluate the expression of mir-486-3p in three groups: PBS, 200 μM H_2_O_2_ and 200 μM H_2_O_2_ + 20 μg/ml ADSC-Exo group. Data represented the mean ± SD of triplicates. ^*^p < *0.05*, ^**^p < 0.01. **F** the differentially expressed genes were subjected to KEGG pathway analysis, which revealed the involvement in the Smad signaling pathway associated EndoMT

To deeply explore the underlying mechanism of aforementioned results, miRNAs-sequences of endothelial cells between Ctrl and H_2_O_2_ group was implemented, as well as between H_2_O_2_ and H_2_O_2_ + Exo group. The transcriptional expression differences of miRNAs were depicted in Fig. 6A-B. The results revealed that there were 55 up-regulated genes and 23 down-regulated genes between Ctrl and H_2_O_2_ group, as well as 44 up-regulated genes and 23 down-regulated genes between H_2_O_2_ and H_2_O_2_ + Exo group. Furthermore, we observed a total of eight common up-regulated intersections and two common down-regulated intersections between both two groups (Fig. 6C). Moreover, as demonstrated in the volcano plot, there was a significant up-regulation of mir-486-3p expression in HUVEC exposed to H_2_O_2._ However, treatment with ADSC-Exo resulted in a notable reduction of its levels (Fig. 6D-E). Meanwhile, we conducted qPCR analysis to validate the sequencing results. As expected, ADSC-Exo effectively ameliorated the up-regulation of miR-486-3p expression in H_2_O_2_-induced HUVEC, exhibiting significant statistical differences between the two experimental groups (Fig. 6F). Additionally, the KEGG pathway analysis revealed a significant correlation between the function of H_2_O_2_/Exo on EndoMT and Smad signaling pathway (Fig. 6G). The transcriptome sequencing and subsequent bioinformatics analysis indicated the potential significance of mir-486-3p in mediating H_2_O_2_-induced EndoMT in HUVEC.

### mir-486-3p facilitated endothelial-to-mesenchymal transition of endothelial cells and directly targeted Sirt6

**Fig. 7:**
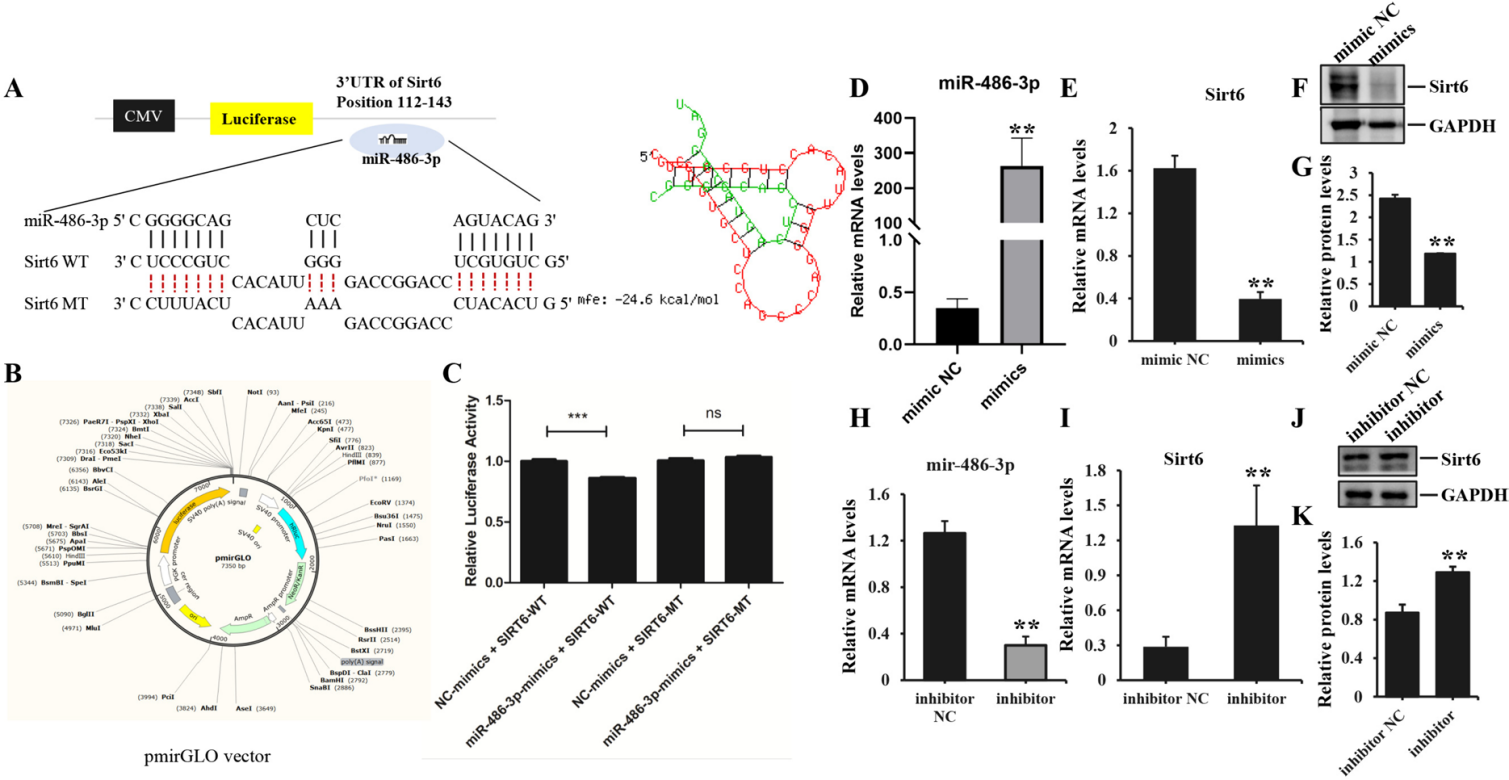
The validation of mir-486-3p in EndoMT. **A** the binding site (position 112–143) of mir-486-3p and the complementary sequences of Sirt6 in the 3'UTR identified through RNAhybrid sequence alignment analysis, which revealed a stem-loop structure. Mir-486-3p was represented in green, while Sirt6 was shown in red. **B** the schematic diagram of the pmirGLO vector for luciferase reporter assays. **C** the luciferase reporter assay revealed the target regulatory relationship between mir-486-3p and Sirt6. ^***^p < *0.001*, *ns*, no significant differences. **D**-**E** the mRNA expression of mir-486-3p and Sirt6 in HUVEC transfected with mir-486-3p mimics was analyzed by qPCR. **F**-**G** immunoblot analysis of the protein expression of Sirt6 in HUVEC transfected with mir-486-3p mimics and the quantitative analysis of protein bands intensity. **H**-**I** the mRNA expression of mir-486-3p and Sirt6 was assessed in HUVEC transfected with mir-486-3p inhibitors. **J**-**K** the protein expression of Sirt6 in HUVEC transfected with mir-486-3p inhibitors and the quantitative analysis of protein bands intensity. Each experiment was conducted in triplicate. Data represented the mean ± SD of triplicates, ^**^p < 0.01

**Fig. 8:**
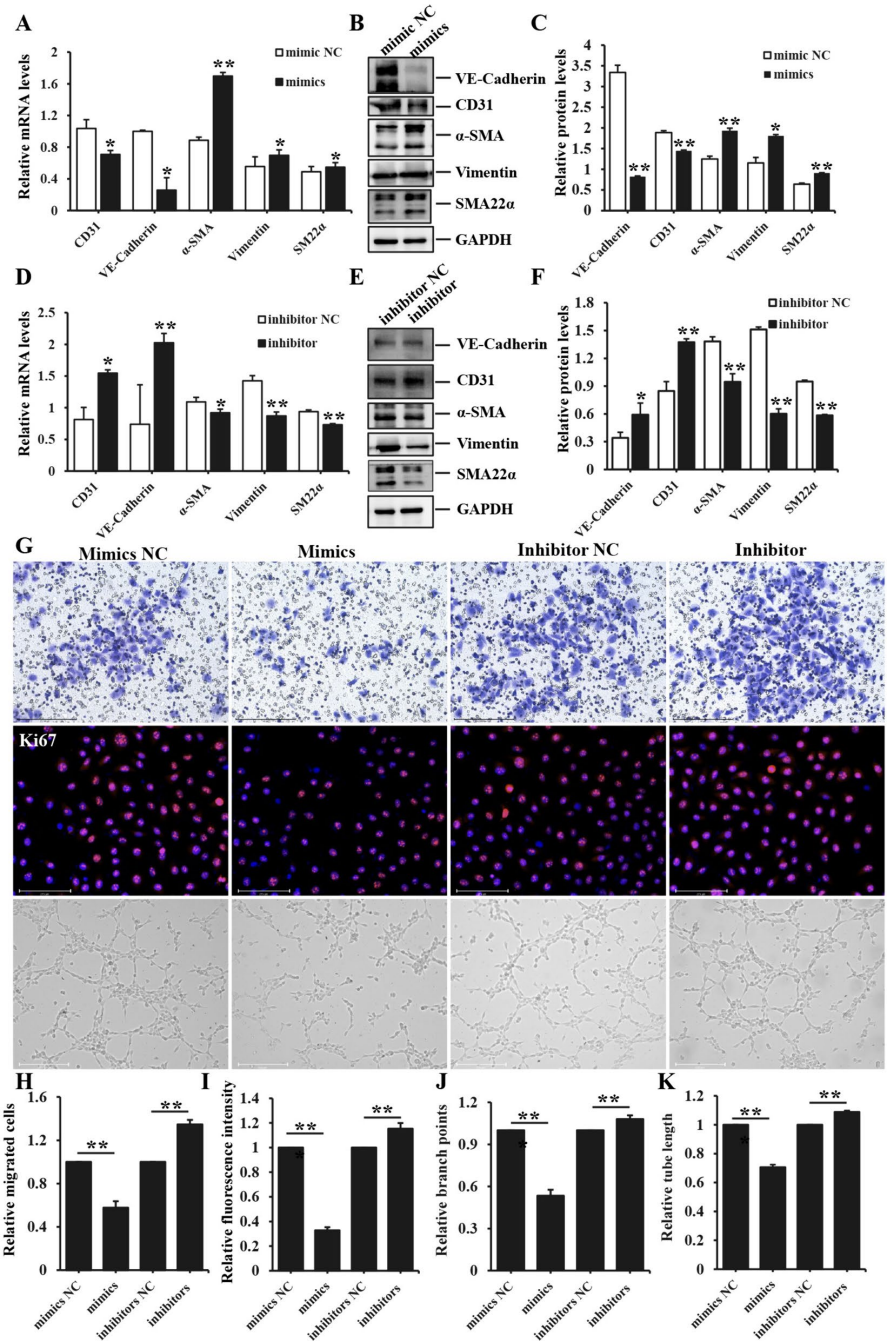
The impact of mir-486-3p on the expression of EndoMT-related markers and the functional characteristics of HUVEC. **A**, **D** qPCR analysis was performed to assess the mRNA expression of CD31, VE-Cadherin, α-SMA, Vimentin and SM22α in HUVEC transfected with mir-486-3p mimics or inhibitors. **B**-**C**, **E**–**F** the protein expression of CD31, VE-Cadherin, α-SMA, Vimentin and SM22α in HUVEC transfected with mir-486-3p mimics or inhibitors was analyzed by immunoblotting and the quantitative analysis of their protein bands intensity were performed by image J software. **G** the effect of mir-486-3p mimics/inhibitors on the proliferation, migration and tube formation of HUVEC. Scale bars = 650 μm, 500 μm and 275 μm. **H** the quantification of migrated cells in transwell assays. **I** the quantitative analysis of mean immunofluorescence intensity of Ki67 in HUVEC exposed to mimics and inhibitors. **J**-**K** the quantity analysis of branch points and tube length in tube formation assays. Each experiment was conducted in triplicate. Data represented the mean ± SD of triplicates. ^*^p < *0.05*, ^**^p < 0.01

The post-transcriptional gene expression is well-known to be repressed by microRNAs through their binding to 3′UTR of target genes. Based on correlation between Sirt6 and fibrosis/Smad signaling pathway, it had been reported that Sirt6 directly interacted with Smad2 in hepatic fibrosis and there was a physical interaction between Sirt6 and Smad3 in hepatic stellate cells. Hence, we subsequently aimed to find the binding sequence of mir-486-3p and Sirt6 through multiple sequence alignment analysis. Additionally, we conducted luciferase reporter assays and performed transfection experiments involving with overexpression or inhibition of mir-486-3p in HUVEC to validate the directly regulatory relationship. The bioinformatics algorithm analysis (RNAhybrid) demonstrated that mir-486-3p exhibited consequential pairing with the target region positions 112–143 of Sirt6 3'UTR, and formed stem-loop structures with certain unconjugated base sequences in Fig. 7A. The luciferase reporter assays illustrated mir-486-3p overexpression significantly reduced luciferase activity when co-transfected with wild-type 3'UTR of Sirt6 (*p≈*0.000026), co-transfection of mir-486-3p with mut-3'UTR of Sirt6 did not exhibit any inhibitory effect on luciferase activity (*p≈*0.209, *ns p* > 0.05) (Fig. 7B-C). Moreover, Sirt6 expression was down-regulated in HUVEC transfected with mir-486-3p mimics, and vice versa (Fig. 7D-K), presenting statistically significant differences between mimics/inhibitor and corresponding negative groups. These findings demonstrated the direct targeting of Sirt6's 3'-UTR by mir-486-3p. Furthermore, mir-486-3p overexpression led to a decrease in CD31 and VE-Cadherin mRNA levels compared to the corresponding negative control, while enhancing the expression of Vimentin, α-SMA and SM22α. Conversely, mir-486-3p inhibition resulted in a pronounced reduction of α-SMA, Vimentin and SM22α expression, and simultaneously increased the mRNA levels of endothelial markers. The protein expression of aforementioned markers was consistent with the corresponding mRNA levels, with notable statistical differences (Fig. 8A-F). Mir-486-3p overexpression effectively suppressed the migration, proliferation, tube formation of HUVEC, showing lesser number of migrated cells and branch points, weaker ki67 fluorescence intensity and shorter tube length with a pronounced significance (Fig. 8G); On the contrary, mir-486-3p inhibition improved biological function of HUVEC, manifesting much number of migrated cell and branch points, stronger ki67 fluorescence intensity and longer tube length with a statistical remarkable difference (Fig. 8H-K). These observations showed mir-486-3p had a regulatory effect on the process of EndoMT in HUVEC.

### H_2_O_2_-induced EndoMT and the therapeutic effect of ADSC-Exo in HUVEC were directly modulated by mir-486-3p

**Fig. 9:**
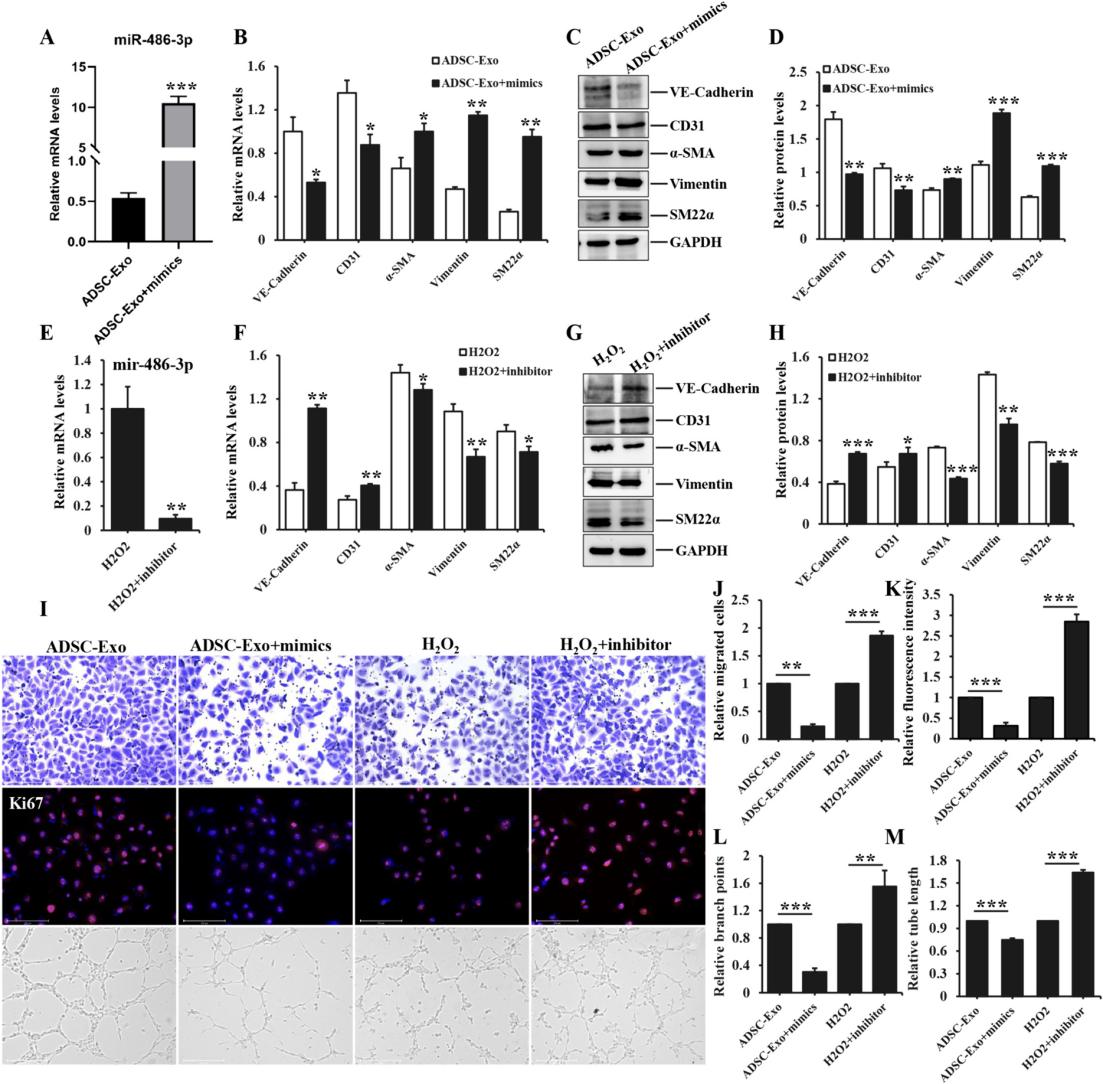
H_2_O_2_-induced EndoMT of HUVEC and the therapeutic effect of ADSC-Exo was directly regulated by mir-486-3p. **A** the expression of mir-486-3p in HUVEC treated with 20 μg/ml ADSC-Exo and 100 nM mir-486-3p mimics. **B** the mRNA expression of fibrotic and endothelial markers in HUVEC stimulated with ADSC-Exo and mir-486-3p mimics. **C** the protein levels of aforementioned markers in HUVEC receiving the same stimulus as described above. **D** the quantitative analysis of WB bands. **E** mir-486-3p levels in HUVEC exposure to 200 μM H_2_O_2_ and 100 nM mir-486-3p inhibitors. **F** the mRNA levels of the related markers of EndoMT in HUVEC treated with the same stimulus as mentioned before. **G** WB analysis of the aforementioned markers. **H** the quantitative analysis of WB bands measured by Image J software. **I** the impact of ADSC-Exo/ADSC-Exo + mir-486-3p mimics and H_2_O_2_/H_2_O_2_ + mir-486-3p inhibitors on the biological function of HUVEC. **J** the quantification of migrated endothelial cells exposure to stimulus as described above in transwell assays. **K** the quantitative analysis of mean immunofluorescence intensity of Ki67 in HUVEC treated with the same interventions. **L**-**M** the quantity analysis of branch points and tube length in tube formation assays. Data represented the mean ± SD of triplicates. ^*^p < *0.05*, ^**^p < 0.01, ^***^ p < 0.0001

To prove the direct regulation of mir-486-3p for EndoMT in H_2_O_2_-induced and ADSC-Exo-intervened HUVEC, we performed the gain-of-function or loss-of-function detection in the following experiments. Mir-486-3p expression was higher in the ADSC-Exo plus mimics group, which verified the successful transfection in Fig. 9A. We further explored the expression of relevant markers of EndoMT, and found the administration of mir-486-3p mimics exacerbated fibrotic markers expression and ameliorated the endothelial markers levels, the difference was significant (Fig. 9B-D). Mir-486-3p overexpression intervention also deteriorated the biological function of HUVEC, manifesting a lesser number of migrated cells and branch points, weaker ki67 fluorescence intensity and shorter tube length (Fig. 9I-M), there were significant differences between ADSC-Exo group and mir-486-3p-concurrent intervention group. Meanwhile, we also found mir-486-3p inhibition could reverse the function of H_2_O_2_-induced EndoMT, revealing the down-regulation of mir-486-3p expression, the increased endothelial markers levels and reduced fibrotic markers expression (Fig. 9D-G), the difference between H_2_O_2_ group and mir-486-3p inhibitors concurrent intervention group had statistical significance. Apart from that, mir-486-3p inhibition restored partly biological function of endothelial cells, showing much number of migrated cells and branch points, stronger ki67 fluorescence intensity and longer tube length, the difference was prominent (Fig. 9I-M). These aforementioned findings illustrated the impact of H_2_O_2_ and ADSC-Exo were directly regulated by mir-486-3p.

### The process of endothelial-to-mesenchymal transition in HUVEC could be inhibited by Sirt6

**Fig. 10:**
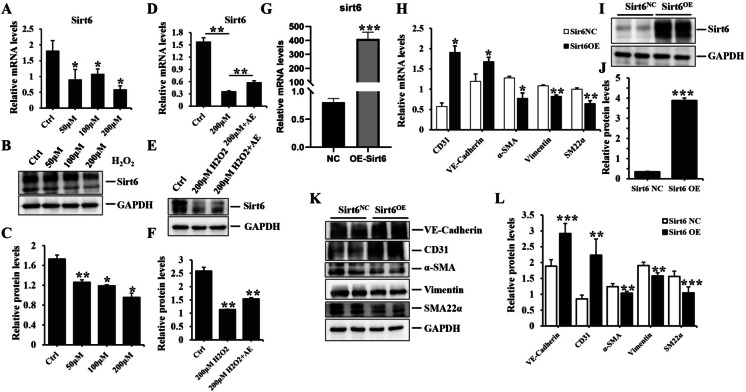
The effect of Sirt6 on EndoMT. **A**-**C** the mRNA and protein expression of Sirt6 in HUVEC exposed to different concentrations of H_2_O_2_ (50 μM, 100 μM or 200 μM) with the quantitative analysis of protein bands intensity. **D**-**F** the mRNA and protein levels of Sirt6 in HUVEC treated with PBS, 200 μM H_2_O_2_ or 200 μM H_2_O_2_ + 20 μg/ml ADSC-Exo group and the quantitative analysis of protein bands intensity. **G**-**H** qPCR analysis of the mRNA levels of CD31, VE-Cadherin, α-SMA, Vimentin and SM22α in HUVEC transfected with either an overexpression plasmid for Sirt6 or a negative control. **I**-**L** immunoblot analysis of the protein levels of CD31, VE-Cadherin, α-SMA, Vimentin and SM22α in HUVEC transfected with either an overexpression plasmid for Sirt6 or a negative control. Data represented the mean ± SD of triplicates. ^*^p < *0.05*, ^**^p < 0.01, ^***^ p < 0.0001

Since mir-486-3p directly modulated Sirt6, the subsequent objective was to investigate the role of Sirt6 in HUVEC exposed to H_2_O_2_ and ADSC-Exo. The results revealed a dose-dependent down-regulation of Sirt6 expression in HUVEC exposed to H_2_O_2_ (50 μM, 100 μM, 200 μM) (Fig. 10A-C), mRNA and protein levels exhibited significant statistical differences between stimulated groups and control group*,* whereas Exo effectively increased Sirt6 expression in HUVEC treated with H_2_O_2_ (Fig. 10D-F), the results exhibited a statistically significant difference between H_2_O_2_ and ADSC-Exo-H_2_O_2_ group_._ More importantly, Sirt6 overexpression facilitated the expression of endothelial markers and suppressed fibrotic markers levels compared to the nontarget group (Fig. 10G-L), with significant statistical differences between Sirt6 overexpression group and negative control group. Consequently, the protective function of Sirt6 antagonizing oxidative stress injury on EndoMT was confirmed.

### ADSC-Exo ameliorated H_2_O_2_-induced EndoMT through the inhibition of Smad signaling pathway

**Fig. 11:**
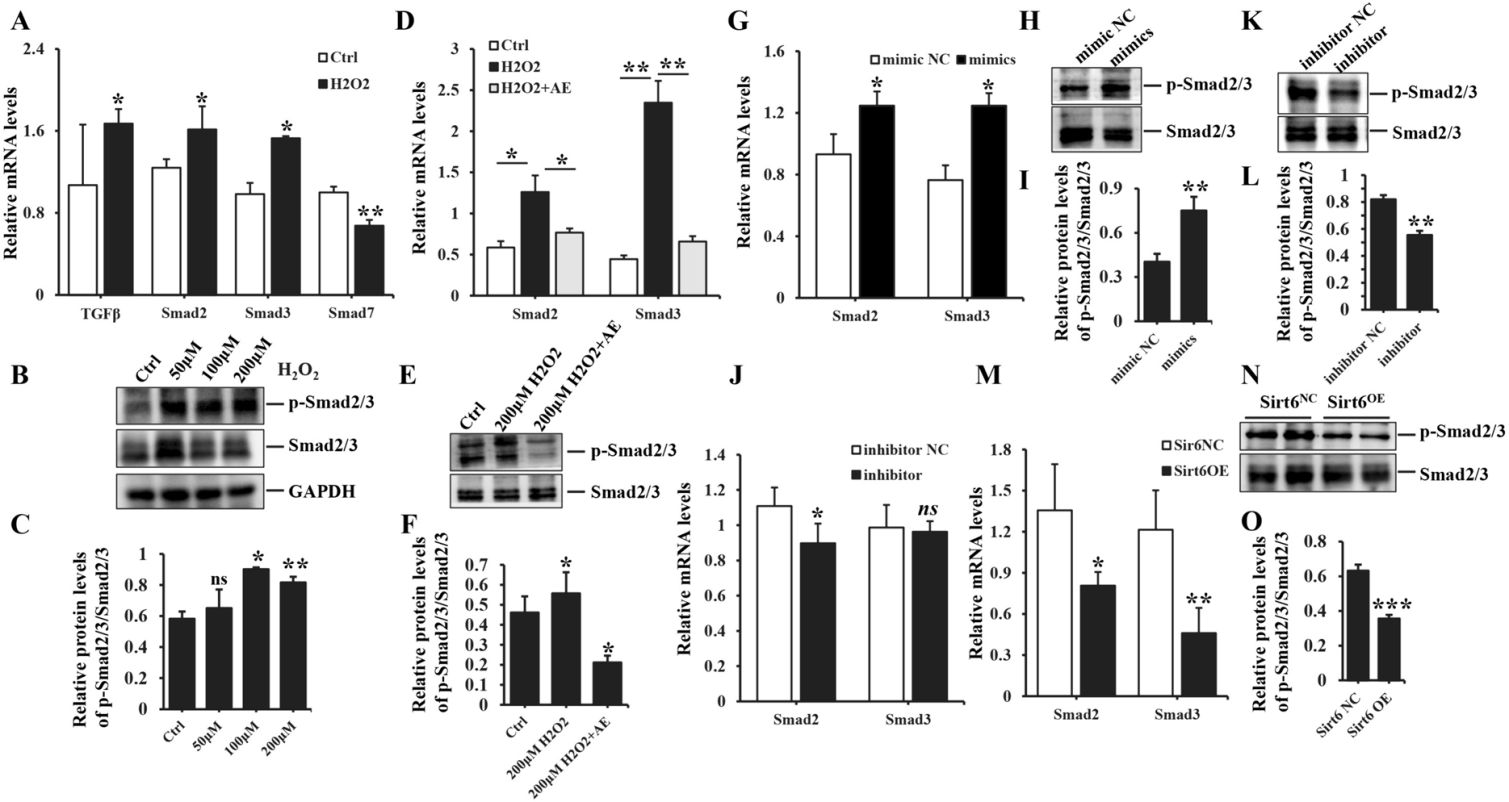
The role of the Smad signaling pathway in H_2_O_2_-induced EndoMT of HUVEC. **A** the mRNA expression of TGFβ1, Smad2, Smad3 and Smad7 in HUVEC exposed to different concentrations of H_2_O_2_ (200 μM). **B**-**C** the protein levels of p-Smad2/3 and Smad2/3 in HUVEC exposure to different concentrations of H_2_O_2_ (50 μM, 100 μM or 200 μM) with the quantitative analysis of protein bands intensity. **D**-**F** the mRNA levels of Smad2 and Smad3, as well as the protein expression of phosphorylated and total Smad2/3, were assessed in HUVEC treated with PBS, 200 μM H_2_O_2_ or 200 μM H_2_O_2_ + 20 μg/ml ADSC-Exo, the quantitative analysis of protein bands intensity was performed by image J software. **G**, **J**, **M** qPCR analysis of the mRNA levels of Smad2 and Smad3 in HUVEC transfected with mir-486-3p mimic or inhibitors, and an overexpression plasmid for Sirt6 or a negative control. **H**-**I**, **K**-**L**, **N**–**O** the protein levels of phosphorylated and total Smad2/3 were assessed in HUVEC transfected with mir-486-3p mimics or inhibitors, and an overexpression plasmid for Sirt6 or a negative control, with the quantitative analysis of protein bands intensity. Data represented the mean ± SD of triplicates. ^*^p < *0.05*, ^**^p < 0.01, ^***^ p < 0.0001

The KEGG analysis revealed that H_2_O_2_-induced EndoMT and the improvement of ADSC-Exo were linked to Smad signaling pathway. We subsequently observed up-regulated phosphorylation and total levels of Smad2/3, as well as increased TGFβ1, Smad2 and Smad3 gene expression in HUVEC exposed to H_2_O_2_. Conversely, mRNA level of Smad7 was reduced. These results presented significant statistical differences between H_2_O_2_ and Ctrl group except for the level of p-Smad2/3/Smad2/3 at 50 μM concentration (Fig. 11A-C). Additionally, phosphorylated Smad2/3 was up-regulated in HUVEC exposure to H_2_O_2,_ whereas ADSC-Exo treatment resulted in a reduction of its expression. Moreover, Smad2 and Smad3 mRNA levels were enhanced in HUVEC exposure to H_2_O_2_ and subsequently attenuated following by ADSC-Exo treatment, with notable statistical differences between the two groups (Fig. 11D-F). Furthermore, p-Smad2/3 and TGFβ1 expression were significantly increased in HUVEC transfected with mir-486-3p mimics, and vice versa, there were also statistically significant differences between mimics/inhibitors and corresponding control groups, except for Smad3 mRNA levels in mir-486-3p inhibitor group (Fig. 11G-L, suppl. Figure 1). Moreover, overexpression of Sirt6 lead to a reduction in phosphorylated Smad2/3, Smad2 and Smad3, the data was statistical difference (Fig. 11M-O). Additionally, TGFβ1 expression was lower in HUVEC transfected with Sirt6-OE plasmid, with a remarkable significance (***p < *0.0001,* suppl. Figure 1). The above findings elucidated that ADSC-Exo effectively attenuated H_2_O_2_-induced EndoMT through mir-486-3p/Sirt6/Smad signaling pathway.

### Overexpression of mir-486-3p could counteract and reverse the beneficial effects of Exo in a murine dorsal wound model

**Fig. 12:**
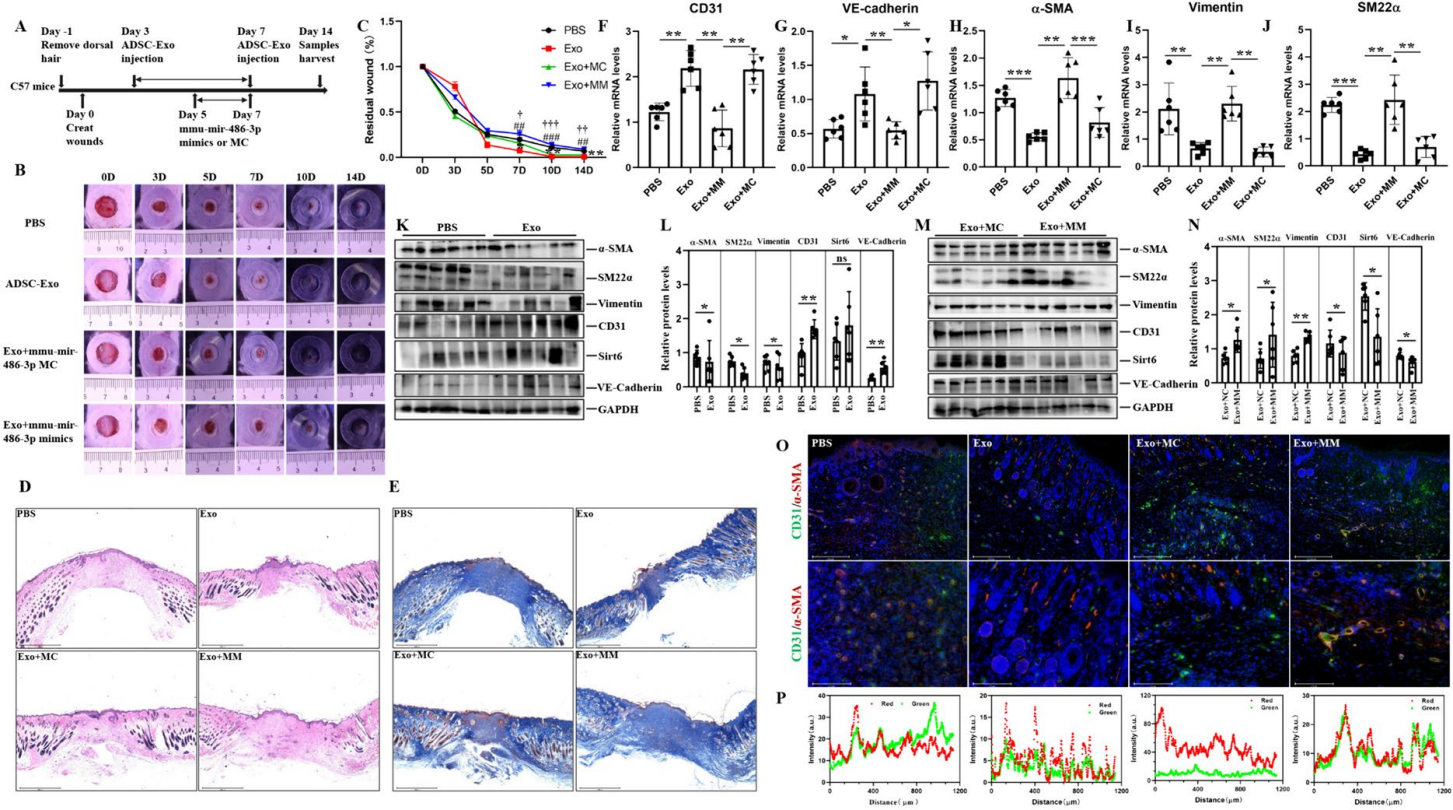
The effect of ADSC-Exo and mir-486-3p were confirmed in a murine wound model. **A** the schematic representation of animal experiments. **B**-**C** the images depicted the temporal evolution of wound morphology at various time points (0D, 3D, 5D, 7D, 10D, 14D)(^*^PBS *vs* Exo, ^*^p < *0.05*, ^**^p < 0.01; ^#^Exo *vs* Exo + Lv-486-3p, ^##^*p* < *0.01*, ^###^*p* < 0.0001; ^†^Exo + Lv-mir-486-3p NC *vs* Exo + Lv-mir-486-3p, ^†^*p* < *0.05,* ^††^*p* < *0.01*, ^†††^*p* < 0.0001). **D**-**E** the murine wound tissues were subjected to routine H&E staining and Masson trichrome staining in PBS group, ADSC-Exo (70 μg/100 μl) group, ADSC-Exo (70 μg/100 μl) plus lentivirus-mediated transfection with mir-486-3p mimics NC (1 × 10^9^TU/ml virus titer in PBS) group, ADSC-Exo (70 μg/100 μl) plus lentivirus-mediated transfection with mir-486-3p mimics groups (1 × 10^9^TU/ml virus titer in PBS). The Masson trichrome staining revealed the presence of a fibrotic region, characterized by a blue coloration. Scale bars = 2000 μm. **F**-**J** the mRNA levels of CD31, VE-Cadherin, α-SMA, Vimentin and SM22α in wound tissues were quantified by qPCR in the aforementioned groups. **K**-**N** western blot analysis was performed to examine the protein expression of Sirt6, CD31, VE-Cadherin, α-SMA, Vimentin and SM22α in wound tissues from different experimental groups as mentioned above with the quantitative analysis of protein intensity measured by Image J software. **O** Immunofluorescence double-labeling staining was performed using antibodies against CD31 (an endothelial lineage marker, green fluorescence) and α-SMA (a fibroblast marker, red fluorescence). Scale bars = 650 μm, 275 μm. **P** the qualitative analysis of colocalization immunofluorescence staining (CD31/green and α-SMA/red) in above mentioned groups. Each group consisted of six mice (n = 6). Data represented the mean ± SD of triplicates. ^*^p < *0.05*, ^**^p < 0.01, ^***^p < 0.0001

**Fig. 13:**
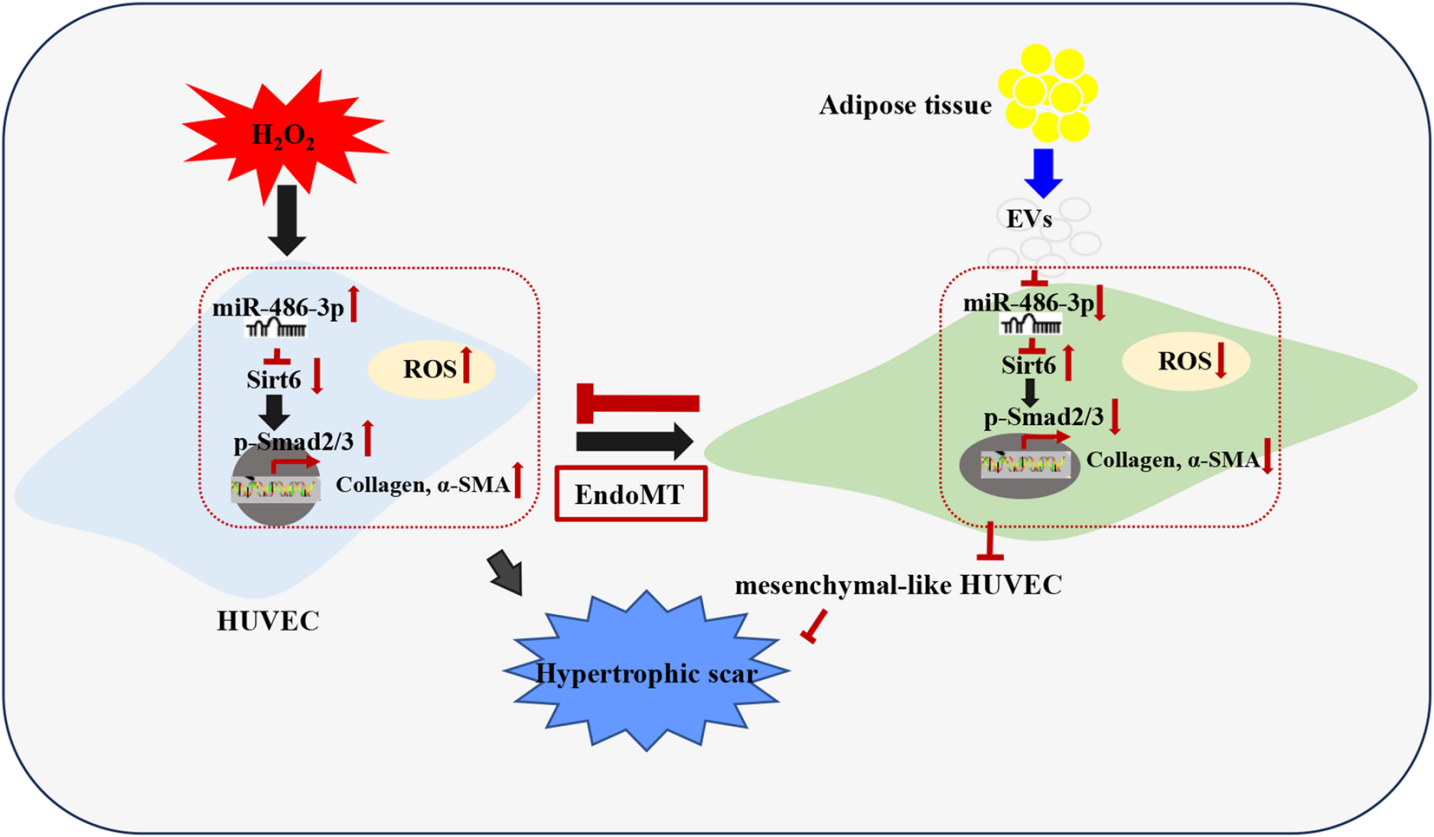
The schematic representation of the study. ADSC-Exo effectively alleviated H_2_O_2_-induced EndoMT and oxidative stress in endothelial cells through the mir-486-3p/Sirt6/Smad signaling pathway

To demonstrate the function of Exo and mir-486-3p in vivo, we further validated the aforementioned results in a murine model with full-thickness skin defects and a strong adhesive silicone ring attached to its periphery to prevent the contraction. The entire experimental procedures were outlined in Fig. 12A. The gross examination demonstrated that ADSC-Exo exhibited a significant capacity to promote wound healing, the rate of wound healing was obviously exacerbated in the lentivirus-mediated transfection of mir-486-3p group, as well as the areas of wounds shown more deterioration, demonstrating statistically significant differences in ratio of wound healing between two groups on Day 7, 10 and 14 (PBS *vs* Exo group, Exo *vs* Exo + Lv-mir-486-3p) (Fig. 12B-C). H&E and Masson trichrome staining of wound tissues samples clearly demonstrated that Exo could accelerate wound healing, alleviate collagen deposition and improve collagen arrangement, resulting in a more organized morphology. mir-486-3p overexpression, however, remarkably exacerbated severity of wound fibrosis, resulting in a more pronounced thickening and irregular arrangement of collagen fibers (Fig. 12D-E). The qPCR analysis revealed an obvious reduction in Vimentin, SM22α and α-SMA expression in Exo group. Conversely, CD31 and VE-Cadherin gene expression were increased in Exo group (Fig. 12F-J), the differences between Exo and the other two groups exhibited statistical significances. Similarly, the expression of CD31, VE-Cadherin and Sirt6 were found to be up-regulated in Exo and Exo + Lv-mir-486-3p NC groups compared to those treated with PBS and Exo + Lv-mir-486-3p groups, respectively. In contrast, the levels of Vimentin, SM22α and α-SMA were down-regulated, also revealing statistically significant differences between PBS and Exo group except for Sirt6 expression, as well as remarkable difference of aforementioned molecules between Exo + mir-486-3p overexpression group and its corresponding control groups (Fig. 12K-N). Although there was a growing tendency for Sirt6 levels after ADSC-Exo treatment, there was probably no statistical differences in the Srt6 expression of murine healed skin tissues due to the significant individual differences between PBS and ADSC-Exo group. In general, ADSC-Exo promoted Sirt6 expression. The colocalization staining revealed that the positive fluorescence intensity of CD31 (green) and α-SMA (red) were predominantly localized in the dermal vasculature of mice in both PBS group and mir-486-3p mimics group (Fig. 12O). Dermal blood vessels lumen in the Exo-treatment group exhibited enhanced distinct, whereas the majority of vessels appeared small, round, and occluded in mir-486-3p overexpression group, a large proportion of fluorescent intension showed the same trends between CD31/red and α-SMA/green in mimics and PBS group (Fig. 12P). These findings provided evidence for the pro-fibrotic effect of mir-486-3p in an in vivo model. Combined with the results of in vitro experiments and the exploration of molecular mechanism as mentioned above, we concluded ADSC-Exo could effectively ameliorate oxidative stress and EndoMT of endothelial cells through mir-486-3p/Sirt6/Smad signaling pathway. (Fig. 13).

## Discussion

Our research provided the following major findings: first of all, we validated the presence of oxidative stress and EndoMT in the dermal vasculature of hypertrophic scar; secondly, we verified the induction of EndoMT and ROS generation in HUVEC treated with H_2_O_2_ through both cell and animal experiments. Endothelial cells underwent transition from a cobblestone-like appearance to a fibroblasts-like shape, while ADSC-Exo exhibited the ability to ameliorate the injury caused by EndoMT and ROS. Furthermore, we identified the differential expression of mir-486-3p in HUVEC exposed to H_2_O_2_ with or without Exo, proved the impact of H_2_O_2_ and Exo on EndoMT of HUVEC, which was directly modulated by mir-486-3p and established a target relationship between mir-486-3p and Sirt6. Additionally, Sirt6 overexpression had been demonstrated to effectively inhibit the process of EndoMT in endothelial cells. The KEGG analysis further revealed a strong correlation between H_2_O_2_-induced EndoMT and the Smad signaling pathway. These collective findings from our experiments had established the theoretical foundation and therapeutic approach for EndMT in hypertrophic scar.

Our discovery regarding the elevated expression of collagen and α-SMA in hypertrophic scar, as well as the increased colocalization of α-SMA and CD31 within the endothelial layer of dermal vasculature in hypertrophic scar, led us to postulate that EndMT might be a significant contributor to hypertrophic scar formation. Our findings were supported by several literatures, indicating that EndoMT was crucial in cardiac fibrosis through changes of microvasculature and extracellular matrix (Zeisberg et al. 2007). The process of EndoMT also led to excessive myofibroblasts activation, resulting in endothelial dysfunction and dermal fibrosis in systemic sclerosis (Manetti et al. 2017), as well as inflammatory response and collagen deposition in pulmonary fibrosis (Li et al. 2022). Furthermore, the amelioration of cell-to-cell tight junction observed by TEM also served as evidence for EndoMT. The stimulation of TGFβ2 was reported to induce YAP dephosphorylation, thereby mediating EndoMT and nuclear transcription in HUVEC through the elevation of ROS levels in subretinal fibrosis (Yang et al. 2022). These findings indicated that the generation of ROS was implicated in the process of EndoMT and fibrosis. Our results elucidated ROS levels were obviously enhanced in hypertrophic scar compared to normal skin and atrophic scar, indicating a potential role of ROS in promoting EndoMT.

The presence of H_2_O_2_ in vitro partially simulated the microenvironment of oxidative stress. The study demonstrated a morphological transition in HUVEC exposed to H_2_O_2,_ characterized by a shift from a cobblestone-like appearance to myofibroblasts-like features (activated *α*-SMA^+^ fibroblasts and collagen secreting), which was referred to as EndMT. This transition was accompanied by reduced levels of endothelial markers and elevated expression of mesenchymal markers, which was consistent with previous reports (Dejana 2017; Li et al. 2018). Meanwhile, we observed an elevation of ROS levels in H_2_O_2_-induced HUVEC. In line with our findings, a study demonstrated that HIF1α-BNIP3-mediated mitophagy possessed the ability to prevent from renal fibrosis by alleviating ROS production (Li et al. 2023). Additionally, another investigation revealed a strong association between ROS activation and CENPA expression as well as the formation of micronuclei in systemic sclerosis fibrosis (Paul et al. 2022). The results of this study validated the induction of EndoMT and oxidative stress in endothelial cells by H_2_O_2_.

Exosomes, serving as a cell-free therapeutic strategy, encompass proteins, metabolites, mRNA, biofluids and nucleic acids for targeted delivery to recipient cells (Baldrick 2023; Park et al. 2020). The literatures had reported that Exo exerted an anti-fibrotic effect by inhibiting EndoMT. MSCs-Exo inhibited EndoMT to ameliorate renal fibrosis in UUO model, and also significantly reduced the right ventricular systolic pressure to suppress the pulmonary vascular remodeling (Choi et al. 2015; Ge et al. 2021). ADSC-Exo exhibited remarkable efficacy in preventing photoaging and inhibiting the UVB-induced ROS accumulation in human dermal fibroblasts (Gao et al. 2021). The exosomal lncRNA SNHG7 derived from MSC also exerted inhibitory effects on EndoMT and vessels generation in diabetic retinopathy through mir34a-5p/XBP1 pathway (Cao et al. 2021). Our findings were consistent with the literatures, indicating that ADSC-Exo effectively reversed the morphological change in HUVEC induced by H_2_O_2_. Additionally, it significantly reduced fibrotic proteins expression (α-SMA, Vimentin and SM22α), and increased endothelial markers levels (CD31 and VE-Cadherin) as well as cellular tight junction protein ZO-1. Meanwhile, ADSC-Exo could mitigate ROS generation and improve biological function in H_2_O_2_-induced HUVEC. These results demonstrated that ADSC-Exo effectively exerted its anti-EndoMT function to repair H_2_O_2_-induced damage in endothelial cells.

MicroRNAs are recognized as the crucial regulators of EndoMT. As for the specific mechanism elucidating the effect of Exo on H_2_O_2_-induced endothelial cells, we could perform the sequencing analysis for exosome (from ADSCs or endothelial cells) or HUVEC with or without H_2_O_2_/ADSC-Exo. Based on the results observed the function of H_2_O_2_ and ADSC-Exo in HUVEC in the aforementioned experiments, we conducted the miRNAs sequencing on endothelial cells stimulated with or without H_2_O_2_/ADSC-Exo. It had been reported that mir-218 derived from human MSC-exosome had the anti-fibrotic properties and inhibited EndoMT through the MeCP2/BMP2 pathway in pulmonary fibrosis (Zhao et al. 2023). The study primarily focused on investigating the influence of mir-486-3p in H_2_O_2_-induced EndoMT of HUVEC and potential improvement of ADSC-Exo through high-throughput sequencing. MiR-486-3p expression had been reported to be increased in patients experienced with liver cirrhosis and mice suffered from liver fibrosis, leading to the regulation of detoxification activity by reduction of UGT1A (Paulusch et al. 2021), and the elevated expression of mir-486 was found in the sera of patients with acute myocardial infarction (Hsu et al. 2014). In the study, we showed an increase of mir-486-3p expression in HUVEC exposed to H_2_O_2_, whereas ADSC-Exo exhibited a mitigating effect on its levels. Additionally, mir-486-3p overexpression was found to promote EndoMT development and inhibit biological function of HUVEC. Likewise, mir-486-3p overexpression exacerbated the beneficial effect of ADSC-Exo, showing aggravated EndoMT and deteriorated the biological function of HUVEC (the capacity of migration, proliferation and tube formation decreased). Mir-486-3p inhibition, on the contrary, reversed the damage caused by EndoMT and restored the biological function of HUVEC, the results demonstrated H_2_O_2_-induced EndoMT and anti-EndoMT of ADSC-Exo were directly modulated by mir-486-3p. The literatures indicated that mir-486 promoted transition of catabolic phenotype in chondrocyte-like cells by directly targeting Sirt6 in patients with severe osteoarthritis (Yang et al. 2021). In the study, the directly regulatory relationship was identified through further bioinformatic analysis, luciferase reporter assays, and gain-of-loss of mir-486-3p function. The subsequent objective was to investigate the impact of Sirt6 on EndoMT in endothelial cells.

The attractiveness of Sirt6 as a therapeutic target had been reported in the conditions of cardiac fibrosis, renal fibrosis, and idiopathic pulmonary fibrosis (Cai et al. 2022; Zhang et al. 2019). The endothelium-specific knockout of Sirt6 induced EndoMT and increased the expression of proinflammatory cytokines in murine carotid arteries (Chen et al. 2021), the involvement of Sirt6-mediated EndoMT in the pathogenesis of diabetic cardiomyopathy had been considered to be critical (Zhang et al. 2020). The results of our study revealed a significant down-regulation of Sirt6 expression in HUVEC exposed to H_2_O_2,_ while treatment with ADSC-Exo was found to effectively increase Sirt6 levels and ameliorate oxidative stress injury induced by EndoMT. Moreover, as a target gene of mir-486-3p, Sirt6 overexpression also conferred protection to endothelial cells against EndoMT and oxidative stress injury by modulating the Smad signaling pathway, thereby indicating the anti-EndoMT effect of Sirt6.

The reduction of H_2_O_2_ in mouse models of cardiac remodeling resulted in a decrease in cardiac fibrosis through the NO-mediated inhibition of phosphorylated Smad (Gee et al. 2022), and the conditioned medium of MSCs partially regulated the Smad pathway to attenuate oxidative stress injury in hepatocytes (Ma et al. 2021). The Sirt6-deficiency fibroblasts exhibited spontaneous trans-differentiation into myofibroblasts, which was attributed to the hyperactivation of TGFβ/Smad signaling, the aberrant activation further led to an induction of fibrosis in mice (Maity et al. 2020). The anti-fibrotic function of Sirt6 was achieved by regulating the TGFβ-Smad2/3 pathway in stellate cells activation as well as liver fibrosis (Zhang et al. 2021). In our study, KEGG analysis illustrated mir-486-3p were modulated by the Smad signaling pathway in HUVEC treated with H_2_O_2_ and ADSC-Exo. The data demonstrated that Smad2 and Smad3 levels, as well as the phosphorylation expression of Smad2/3, were significantly elevated in HUVEC treated with H_2_O_2_ or overexpression of mir-486-3p. This suggested that both H_2_O_2_ and mir-486-3p had the ability to activate the Smad signaling pathway. Conversely, ADSC-Exo treatment, mir-486-3p inhibition, or Sirt6 overexpression effectively alleviated phosphorylated Smad2/3 protein expression in HUVEC. More importantly, we confirmed the influence of Exo and mir-486-3p overexpression on animal models, indicating Exo boosted wound healing and the expression of endothelial markers, inhibited collagen deposition and the levels of fibrotic markers, suppressed the occurrence and development of EndoMT in dermal vessels, resulting in rapid wound healing and lesser fibrotic remodeling. Conversely, mir-486-3p overexpression exacerbated the fibrosis and EndoMT to delay wound healing.

## Conclusions

These findings suggested that ADSC-Exo exerted cytoprotective effects against H_2_O_2_-induced EndoMT in endothelial cells by inhibiting the mir-486-3p/Sirt6/Smad signaling pathway. The study simultaneously provided the theoretical foundation for elucidating the effect of oxidative stress on endothelial-mesenchymal transition in dermal vasculature of hypertrophic scar. The therapeutic effects of ADSC-Exo and mir-486-3p inhibition were also observed, indicating their potential for further clinical applications.

## Supplementary Information

Below is the link to the electronic supplementary material.

Supplementary file1 (DOCX 146 KB)

## Data Availability

Not applicable.

## Abbreviations

NS: Normal skin
HS: Hypertrophic scar
AS: Atrophic scar
α-SMA: α-Smooth muscle actin
Col1: Type I Collagen
Col3: Type III Collagen
ADSCs: Adipose tissues derived stem cells
ADSC-Exo: ADSCs derived exosome
DMEM: Dulbecco′s modified Eagle′s medium
FBS: Fetal bovine serum
DAPI: 4′, 6-Diamidino-2-phenylindole
GAPDH: Glyceraldehyde-3-phosphate dehydrogenase
BSA: Bovine serum albumin
TEM: Transmission electron microscope
NTA: Nanoparticle tracking analysis
3′UTR: 3′-Untranslated regions
Sirt6: Silencing information regulator-sirtuin6
Smad2: Mothers against decapentaplegic homolog 2
H_2_O_2_: Hydrogen peroxide
ROS: Reactive oxygen species
EndoMT: Endothelial-to-mesenchymal transition
HUVECs: Human umbilical vein endothelial cells
