# Supplementary material for: Exosomes derived from adipose tissue-derived stem cells alleviated H2O2-induced oxidative stress and endothelial-to-mesenchymal transition in human umbilical vein endothelial cells by inhibition of the mir-486-3p/Sirt6/Smad signaling pathway
Source: Cell Biol Toxicol. 2024 May 25;40(1):39. doi: 10.1007/s10565-024-09881-6 (PMC11126451; doi:10.1007/s10565-024-09881-6)
Supplement: Supplementary file 1 — Supplementary file1 (DOCX 146 KB) [file 10565_2024_9881_MOESM1_ESM.docx]

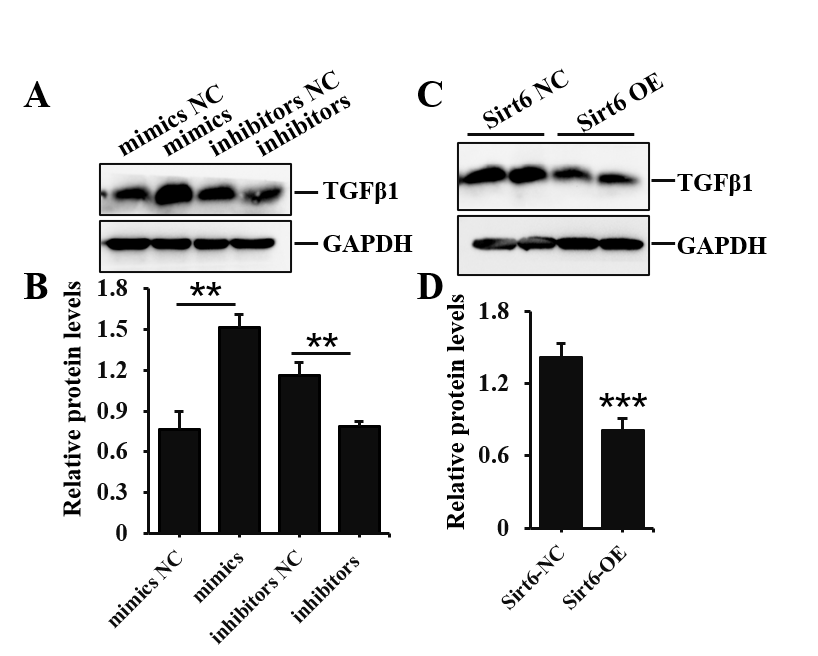


Suppl. Fig1

**The effect of mir-486-3p and Sirt6 on TGFβ1 expression.** (A) WB analysis of TGFβ1 expression in HUVEC stimulated with mir-486-3p mimics, inhibitors and their corresponding negative control. (B) the quantitative analysis of TGFβ1 levels measured by Image J software. (C) the expression of TGFβ1 in HUVEC treated with Sirt6-OE plasmid detected by western blotting. (D) quantity analysis of WB bands. Data represented the mean ± SD of triplicates. ^**^*p*< 0.01, ^***^*p* < 0.001.
